# Transcriptomic analysis of the temporal host response to skin infestation with the ectoparasitic mite *Psoroptes ovis*

**DOI:** 10.1186/1471-2164-11-624

**Published:** 2010-11-10

**Authors:** Stewart TG Burgess, David Frew, Francesca Nunn, Craig A Watkins, Tom N McNeilly, Alasdair J Nisbet, John F Huntley

**Affiliations:** 1 Moredun Research Institute, Pentlands Science Park, Bush Loan, Edinburgh, Midlothian, EH26 0PZ, Scotland, UK

## Abstract

**Background:**

Infestation of ovine skin with the ectoparasitic mite *Psoroptes ovis *results in a rapid cutaneous immune response, leading to the crusted skin lesions characteristic of sheep scab. Little is known regarding the mechanisms by which such a profound inflammatory response is instigated and to identify novel vaccine and drug targets a better understanding of the host-parasite relationship is essential. The main objective of this study was to perform a combined network and pathway analysis of the *in vivo *skin response to infestation with *P. ovis *to gain a clearer understanding of the mechanisms and signalling pathways involved.

**Results:**

Infestation with *P. *ovis resulted in differential expression of 1,552 genes over a 24 hour time course. Clustering by peak gene expression enabled classification of genes into temporally related groupings. Network and pathway analysis of clusters identified key signalling pathways involved in the host response to infestation. The analysis implicated a number of genes with roles in allergy and inflammation, including pro-inflammatory cytokines (*IL1A, IL1B, IL6, IL8 *and *TNF*) and factors involved in immune cell activation and recruitment (*SELE, SELL, SELP, ICAM1, CSF2, CSF3, CCL2 *and *CXCL2*). The analysis also highlighted the influence of the transcription factors NF-kB and AP-1 in the early pro-inflammatory response, and demonstrated a bias towards a Th2 type immune response.

**Conclusions:**

This study has provided novel insights into the signalling mechanisms leading to the development of a pro-inflammatory response in sheep scab, whilst providing crucial information regarding the nature of mite factors that may trigger this response. It has enabled the elucidation of the temporal patterns by which the immune system is regulated following exposure to *P. ovis*, providing novel insights into the mechanisms underlying lesion development. This study has improved our existing knowledge of the host response to *P. ovis*, including the identification of key parallels between sheep scab and other inflammatory skin disorders and the identification of potential targets for disease control.

## Background

Sheep scab, caused by the mite *Psoroptes ovis *is, arguably, the most important ectoparasitic disease of sheep in the UK.The disease is highly contagious, causing pruritis and irritation and is a major welfare concern [1]. Current disease control strategies are reliant upon chemotherapy; however concerns over residues, eco-toxicity and parasite resistance have raised concerns regarding current control strategies [2]. Developing alternative control methods requires a deeper understanding of both the parasite and its interaction with the host.

The life cycle of *P. ovis *is carried out on the ovine host and takes from 11-19 days from egg hatch to egg production by the adult [3]. Mites can survive off host, but only remain infective for 15-16 days once removed from the skin [4]. *P. ovis *is a non-burrowing, surface exudate feeder capable of consuming serous fluids, lymph and red blood cells [5]. Mites survive on the surface of the skin and their mouthparts do not appear to penetrate beyond the stratum corneum [6]. The available evidence suggests that mites abrade the stratum corneum, depositing allergens as they progress and this combination of skin abrasion, allergen deposition and self-grooming behaviour by the host in response to the pruritis caused by the mites triggers the subsequent activation of a cutaneous inflammatory response [7] including an exudate which supplies the mite with a food source [8]. Terminally differentiated keratinocyte cells, termed corneocytes, within the stratum corneum are therefore the first point of contact between the parasite and the host immune response. Establishment of a *P. ovis *infestation is the result of a complex interaction between host and mite, during which the mite appears to initiate reactions conducive to its own establishment and maintenance [9]. The skin lesions are induced by mite-derived pro-inflammatory factors, a likely source of which, are the mite excretory/secretory products, including potent enzymes and allergens (reviewed in [8]). While several mite products have been identified, including a number of enzymes and homologues of allergens of the house dust mite (HDM), *Dermatophagoides pteronyssinus*[10].

A major feature of sheep scab is the rapid epidermal influx of eosinophils and neutrophils, followed by blister formation and a serous fluid exudate resulting in dermal oedema [11]. The sheep scab lesion is histopathologically consistent with those described for allergic dermatitis [12]. IgE-mediated immediate hypersensitivity responses may participate in the lesion pathology or immunity at later stages of infestation; however the initial process of establishment involving pro-inflammatory responses is unlikely to involve IgE-mediated reactions, since this occurs in mite-naïve sheep. The mechanism of this early pro-inflammatory response is unknown, but represents a pivotal step in mite colonization and is critical in determining disease progression. Prior infestation with sheep scab alters progression of subsequent infestations with reduced lesion size and mite numbers in secondary infestations [13]. This evidence offers encouragement for control by vaccination [2].

Our group recently demonstrated the up-regulation of the pro-inflammatory factor interleukin-8 (*IL8*) in ovine keratinocytes within 1 hour of *P. ovis *exposure, supporting the rapidity and pro-inflammatory nature of the host response [14]. Here, in order to characterise the host skin response during the first 24 hours post-infestation we employed an ovine transcriptome microarray. Clustering, network and pathway mapping approaches provided new insights into the key signalling events instigating the pro-inflammatory response and leading to lesion development. This study has elucidated temporal patterns by which the immune system is regulated and has revealed information regarding the nature of the mite factors that trigger the pro-inflammatory response.

## Results & Discussion

### Total RNA extraction from skin biopsy samples

Total RNA extracted from skin biopsies was assessed for quality based on the RNA Integrity Number (RIN) and quantified using a spectrophotometer [15]. High quality total RNA was extracted from all skin biopsies; non-infected biopsy samples showed a mean RIN of 9.7 (minimum RIN = 9) and a mean yield of 52 μg from 30-50 mg tissue biopsy (0.1-0.173% starting material). Infected biopsy samples showed a mean RIN of 9.6 (minimum RIN = 8.8) and a mean yield of 70 μg from 30-50 mg tissue biopsy (0.14-0.23% of starting material). It was noted that the skin within the chambers became visibly reddened within 30 minutes of infestation with *P. ovis *mites indicating a rapid pro-inflammatory response.

### Microarray data processing

To ensure quality and consistency of sample labelling and array hybridizations, control information was collated from all arrays and reviewed prior to data analysis. Quality control data for all arrays was found to be consistent with the manufacturer's (Agilent) recommendations. The performance of the array hybridizations was further assessed through scatter plots, comparing each array with every other generated, in Genespring GX 11.0. Scatter plots confirmed a linear distribution between arrays and showed a dynamic uninterrupted range of expression values from low to high signals. Box and whisker visualizations confirmed the data had comparable distributions and were of sufficient quality for further analysis. To remove invariant transcripts that could contribute to multiple testing errors in the subsequent statistical analysis, downstream data filtering of the array dataset (15,208 probes) was performed. Probes showing a "present" or "marginal" flag call in 100% of the samples in any one of the 5 time points were considered reliably expressed (13,517 probes). This list was further filtered on expression values, retaining probes with 100% of the samples in any one of the 5 time points showing expression values within the 20-100^th ^percentile. These filtering steps resulted in a final list of 10,716 probes for the differential expression analysis.

### Determination of differentially expressed transcripts

**Figure 1:**
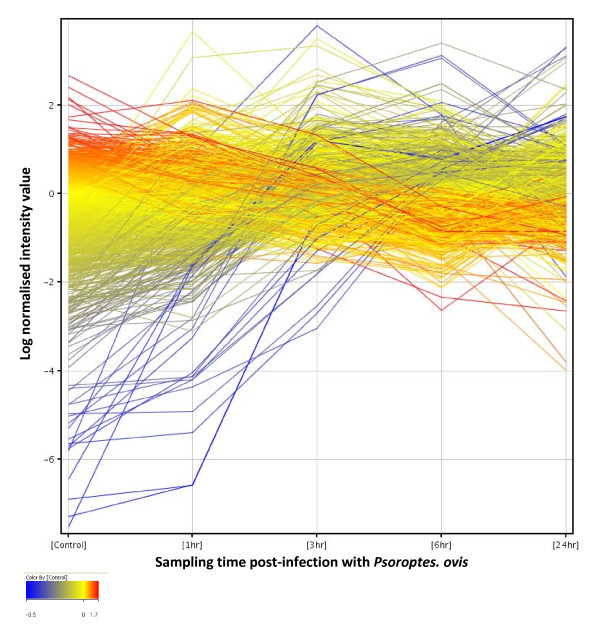
**Profile plot of 1,552 genes significantly differentially expressed during time course of infestation with *P. ovis***. Gene expression profiles for differentially expressed genes over the time course of infestation with *P. ovis*. Control (Time = 0), 1 hour, 3 hours, 6 hours and 24 hours post-infestation. Each line represents a single gene, colour coded by log normalised intensity values

Infestation of the skin with *P. ovis *mites results in a rapid pro-inflammatory response within minutes of contact [16]. As such we focused our analyses on the first 24 hours post infestation to elucidate the signalling pathways involved in disease development. 1,552 genes were found to be significantly differentially expressed in at least one of the 10 possible time point comparisons [non-infected control (C) *vs *1 hour post infestation (hpi), C vs 3hpi, C vs 6hpi, C vs 24hpi, 1hpi vs 3hpi, 1hpi vs 6hpi, 1hpi vs 24hpi, 3hpi vs 6hpi, 3hpi vs 24hpi and 6hpi vs 24hpi] (Figure 1). Multiple test correction was performed using the Benjamini & Hochberg False Discovery Rate (FDR) procedure with an FDR corrected p-value cut-off set at ≤ 0.05, therefore 5% of genes could be expected to pass this filtering step by chance and represent potential false positives (77 genes). Of the 1,552 probes, gene symbol level annotation was available for 1,383 probes (89%); the relevant homologous human gene symbol was used where the ovine annotation was unavailable. This annotation was obtained either from the Agilent ovine gene expression microarray annotation data or from individual BLAST analysis of the EST sequences represented by each probe, leaving 169 probes (11%) for which no annotation was available. These probes were excluded from the network/pathway analysis described below, leaving 1,383 annotated probes available. The updated annotation of the Agilent ovine gene expression microarray is available from the authors on request. Protocols of the experimental procedures, methods of analysis and microarray data are available as supplementary information in the European Bioinformatics Institute's ArrayExpress database http://www.ebi.ac.uk/arrayexpress accession number E-TABM-1012.

### Quantitative real time PCR (qRT-PCR) validation of microarray data

To confirm the validity of the microarray results, qRT-PCR confirmation was undertaken for 11 putative differentially expressed genes (Table 1). Differential expression of selected genes was considered to be validated as the fold change data showed a mean correlation co-efficient of 0.87 between the qRT-PCR and microarray data. In addition, interleukin 4 (*IL4*) was significantly differentially expressed following infestation with a 9-fold increase compared to control samples at 3 and 6 hpi (p = 0.05) falling back to baseline levels at 24 hpi. *CSF2 *was also significantly differentially expressed (p = < 0.01) with a 9-10 fold increase compared to control samples at 1, 3 and 6 hpi again falling back to baseline levels by 24 hpi.

**Table 1:** qRT-PCR validation of microarray data

Gene Symbol	Microarray fold change				qRT-PCR fold change				Correlation*
	1 hr	3 hr	6 hr	24 hrs	1 hr	3 hr	6 hr	24 hrs	
*IL1B*	4.9	14.6	5.7	1.7	41.0	127.4	52.6	5.2	0.99
*IL6*	103.3	771.7	193.0	15.2	6.4	11.9	6.1	1.4	0.92
*IL8*	44.0	311.9	231.6	454.6	21.7	244.5	293.8	477.4	0.96
*TNF*	3.4	1.3	1.5	1.3	5.2	2	2.5	0.8	0.95
*IL10*	2.2	2.1	2.8	2.3	1.8	1.7	1.9	0.7	0.4
*IL18*	1.1	1.1	1.4	1.3	1.4	1.3	1.6	0.7	0.68
*TGFBI*	1.5	1.3	1.5	3.2	1.6	1.7	1.7	3.2	0.99
*TLR4*	1.3	2.3	2.0	1.2	0.8	1.5	1.2	0.5	0.96
*TLR2*	2.0	4.6	3.4	2.8	1.5	2.7	2.3	1.0	0.83
*IL4*	4.2	19.5	28.8	2.2	1.9	8.7	9.2	0.8	0.97
*CSF2*	6.7	4.9	4.4	1.6	9.2	9.1	9.8	1.4	0.86
							*Mean correlation*		*0.87*

Key - Comparisons between baseline samples (time = 0) and individual time points. *Correlations performed using Pearson's correlation co-efficient for highly expressed genes, i.e. *IL1B, IL6, IL8, TNF, TLR4, TLR2, IL4 *and *CSF2 *and Spearman's rank correlation co-efficient for genes with lower expression levels, i.e. *IL10, IL18 *and *TGFBI*

### Temporal modulation of the host inflammatory response

**Figure 2:**
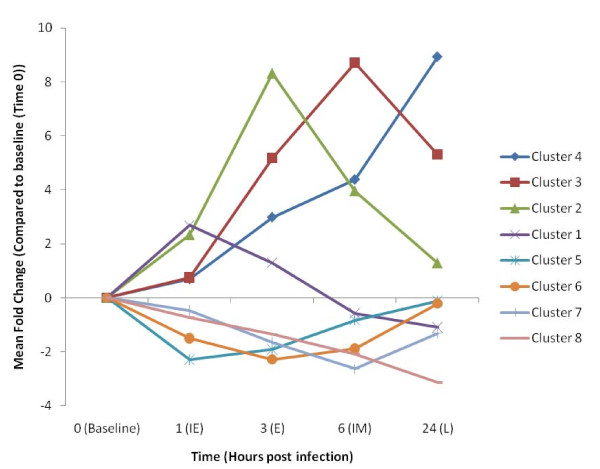
**Clustered mean temporal gene expression profiles following infestation with *P. ovis***. Mean gene expression profiles for gene expression clusters 1-8. Time (hours) post infestation highlighted on X-axis and the mean fold change compared to time zero sample on the Y-axis. Baseline = non-infected, IE = immediate early, E = early, IM = intermediate and L = late

All significantly differentially expressed genes were grouped into distinct clusters based on the time point (post-infestation) at which their expression level peaked. This produced eight distinct gene clusters with defined temporal peaks of expression across the time course of infestation (Table 2). To aid data visualisation, mean expression profiles were calculated to represent the individual gene clusters. These profiles were calculated using the mean fold change profile of all genes in a cluster over the time course of infestation, as compared to the baseline (Time = 0) sample data and highlight the temporal groupings of genes over the first 24 hours of infestation (Figure 2). The individual gene clusters could be further classified based on their mean temporal pattern of expression, i.e. genes induced within 1 hour [Immediate Early (IE)]; induced within 3 hours [Early (E)]; induced within 6 hours [Intermediate (IM)] and those genes induced across the 24 hour time course [Late (L)]. The same nomenclature was also applied to the repressed genes (Table 2). The gene lists for the individual clusters are provided in Additional File 1. The genes clustered well based on the time of peak expression however we noted further similarities in the temporal pattern of genes in each group. For example genes in cluster 1 showed a peak of expression at 1 hpi and then quickly declined back to baseline levels (or below) by 6 hpi. Genes in cluster 2 showed increased expression until 3 hpi and then dropped back towards baseline levels by 24 hpi. The level of expression of the genes in cluster 3 increased to a maximum at 6 hpi and then tapered off by 24 hpi, whilst genes in cluster 4 demonstrated a steady increase in expression over the 24 hour time course (Figure 2). Similar patterns were observed for the repressed genes (Figure 2). The process of clustering genes based on the time of their peak expression over the time course of infestation with *P. ovis *represented a successful method of grouping genes and allowed the investigation of the temporal patterns of gene expression in infected skin to be further analysed.

**Table 2:** Peak temporal gene expression clusters following infestation with *P. ovis*

Cluster ID	Cluster Description	Number of genes in cluster (% of total)
1	Immediate early (IE) up	81 (5.2)
2	Early (E) up	201 (12.9)
3	Intermediate (IM) up	344 (22.2)
4	Late (L) up	383 (24.7)
5	Immediate early (IE) repressed	6 (0.4)
6	Early (E) repressed	35 (2.3)
7	Intermediate (IM) repressed	289 (18.6)
8	L (L) repressed	213 (13.7)

### Comparison of the host response to *P. ovis *infestation with house dust mite allergy pathogenesis

To identify key pathways involved in the host skin response to infestation with *P. ovis *we compared our dataset (1,383 annotated probes) with a dataset of 315 genes known to be differentially expressed in human primary nasal epithelium following exposure to HDM extract [17]. This comparison identified 114 genes (36%) from the HDM dataset that were also differentially expressed in our dataset, indicating a core, common response to mite allergen exposure from two Astigmatid species at two distinct tissue sites (skin and nasal epithelium). The majority of these common differentially expressed genes were up-regulated (90/114, 79%) and most showed peak expression between 1-3 hpi. This list showed enrichment for factors involved in "cellular growth and proliferation" (n = 55), "gene expression" (n = 58), "inflammatory disease" (n = 41) and "dermatological diseases and conditions" (n = 23). There was also enrichment for the "acute phase response signalling" (p = 3.8E^-12^), "IL10" (p = 2.3E^-11^) and "IL6" (p = 7.2E^-10^) canonical signalling pathways. We next compared our dataset against a list of 511 common host response genes identified by Jenner *et al*., [18] in a meta-analysis of multiple microarray datasets investigating human host responses to multiple pathogens in different cell types. This comparison showed that 189 of the 511 host response genes (36%) were differentially expressed following infestation with *P. ovis*, indicating the presence of a strong host immune response to infestation. Of these 189 genes, 169 (89%) were up-regulated following infestation with *P. ovis *and the time at which these genes showed peak expression was spread across the time course of infestation with 15 genes up-regulated at 1 hpi, 44 genes at 3 hpi, 72 genes at 6 hpi and 39 genes at 24 hpi. In contrast, 17 of the 20 down-regulated genes showed a peak repression in their expression level at 24 hours. These temporal patterns of expression point to a common host response to infestation with *P. ovis *with the highest activity in immune response genes between 3 and 24 hpi. The list of common host response genes showed enrichment for factors involved in "inflammatory response" (n = 76), "cellular growth and proliferation" (n = 98) and "cellular movement" (n = 67) and significant enrichment for the "dendritic cell maturation" (p = 7.04E^-15^), "communication between innate and adaptive immune cells" (p = 2E^-14^), "IL10" (p = 2.48E^-13^), "IL6" (p = 1.26E^-12^), "TREM1" (p = 1.04E^-10^), "NF-kB" (p = 9E^-10^) and "T helper cell differentiation" (p = 2.56E^-06^) signalling pathways.

### Network/Pathway analysis of temporal host response to *P. ovis *infestation

The differential regulation of individual genes does not necessarily relate to the importance of a biological effect as these changes can also be due to indirect or bystander effects. This can be a particular problem when these changes are analysed in isolation. To limit the impact of these effects we utilised a network/pathway approach, analysing global changes in gene expression in the context of known signalling pathways, enabling the identification of functional groupings of genes. To identify the temporal mechanisms and signalling pathways involved in the host response to *P. ovis *infestation we undertook a network/pathway based analysis of clusters 1-8, representing all 1,383 annotated differentially expressed genes from across the time course of infestation (Figure 2). The 10 genes with the highest greatest fold change compared to baseline from each of the clusters are shown in Table 3 and the top five canonical signalling pathways from the Ingenuity Pathway Analysis (IPA) mapping are documented in Table 4.

**Table 3:** Top 10 fold-changing genes from each temporal cluster following infestation with *P. ovis*

Cluster ID	Gene Symbol	Gene Description	Fold Change*
***Cluster 1***	*TMEM123*	Transmembrane protein 123	19.3
	*CD83*	CD83 molecule	9.1
	*ATF3*	Activating transcription factor 3	7.5
	*CYR61*	Cysteine-rich, angiogenic inducer, 61	5.4
	*BTG2*	BTG family, member 2	5.3
	*EGR1*	early growth response 1	4.5
	*CYR61 (2×)*	cysteine-rich, angiogenic inducer, 61	4.5
	*GPR171*	G protein-coupled receptor 171	4.4
	*GADD45B*	growth arrest and DNA-damage-inducible, beta	4.1
***Cluster 2***	*IL6*	Interleukin-6	771.7
	*CCL2*	Chemokine (C-C motif) ligand 2	35.7
	*CXCL2*	Chemokine (C-X-C motif) ligand 2	22.7
	Unknown	N/A	19.1
	*SELP (2×)*	Selectin P (granule membrane protein 140 kDa, antigen CD62)	17.2 to 18.1
	*SOCS3*	Suppressor of cytokine signalling 3	18.0
	*CSF3*	colony stimulating factor 3 (granulocyte)	17.6
	*PDE4B*	phosphodiesterase 4B, cAMP-specific (phosphodiesterase E4 dunce homolog, Drosophila)	17.1
	*TNFAIP6*	tumor necrosis factor, alpha-induced protein 6	16.7
***Cluster 3***	*PTX3 (2×)*	Pentraxin 3	225.6 to 317.2
	*MMP1 (2×)*	Matrix metallopeptidase 1 (interstitial collagenase)	237.1 to 302.8
	*CXCL5*	Chemokine (C-X-C motif) ligand 5	218.0
	*SDS*	Serine dehydratase	134.3
	*MT1E*	Metallothionein 1E	76.9
	*PLEK*	pleckstrin	48.8
	*MS4A8B*	membrane-spanning 4-domains, subfamily A, member 8B	45.2
	*SOD2*	superoxide dismutase 2, mitochondrial	31.5
***Cluster 4***	*IL8 (2×)*	Interleukin-8	291.7 to 617.4
	*SPRR2A*	Small proline-rich protein 2A	324.5
	*CSN2*	Casein beta	174.7
	*SPRR2E (2×)*	small proline-rich protein 2E	109.7 to 163.2
	*LTF*	Lactotransferrin	121.9
	*S100A9*	S100 calcium binding protein A9	90.9
	*ASGR2*	asialoglycoprotein receptor 2	66.4
	*S100A12*	S100 calcium binding protein A12	66.3
***Cluster 5^#^***	*APOBEC2*	Apolipoprotein B mRNA editing enzyme, catalytic polypeptide-like 2	-4.6
	Unknown	N/A	-2.1
	*ZACN*	Zinc activated ligand-gated ion channel	-2.0
	*HSPA8*	Heat shock 70 kDa protein 8	-1.7
	*ADAMTS2*	ADAM metallopeptidase with thrombospondin type 1 motif, 2	-1.7
	*FGL2*	Fibrinogen-like 2	-1.6
***Cluster 6***	*SFRP4*	Secreted frizzled-related protein 4	-5.0
	*TXNIP (3×)*	Thioredoxin interacting protein	-3.4 to -3.9
	Unknown	N/A	-2.8
	*SERPINF1*	Serpin peptidase inhibitor, clade F, member 1	-2.7
	*FZD2 (2×)*	Frizzled homolog 2	-2.6 to -2.7
	*C3*	Complement component 3	-2.4
	*FCGBP*	Fc fragment of IgG binding protein	-2.3
***Cluster 7***	*SDPR*	Serum deprivation response	-19.7
	*NOSTRIN*	nitric oxide synthase trafficker	-6.6
	*FAM13C*	Family with sequence similarity 13, member C	-5.8
	*CDKN1C*	Cyclin-dependent kinase inhibitor 1C (p57, Kip2)	-5.8
	Unknown	N/A	-5.6
	*LIX1L*	Lix1 homolog (mouse)-like	-5.5
	*SCARA5*	Scavenger receptor class A, member 5 (putative)	-5.3
	*COL3A1*	collagen, type III, alpha 1	-4.9 to -5.1
***Cluster 8***	*CYP2F1*	Cytochrome P450, family 2, subfamily F, polypeptide 1	-29.2
	*CAP2*	CAP, adenylate cyclase-associated protein, 2	-28.5
	*LOR*	Loricrin	-26.3
	*IGFBP1*	Insulin-like growth factor binding protein 1	-23.4
	*STMN2*	Stathmin-like 2	-12.9
	*GFRA2*	GDNF family receptor alpha 2	-11.5
	*CYP2B*	Cytochrome P450, family 2, subfamily B	-9.3
	*FLG*	Filaggrin	-8.6
	*KRT1*	Keratin 1	-7.0
	*FMO3*	Flavin containing monooxygenase 3	-6.4

Key - *Fold change calculated relative to baseline samples (Time = 0), range given where 2 or more probes for a gene are present. #Cluster 5 contained only 6 genes

**Table 4:** Top five canonical pathways associated with each gene cluster following infestation with *P. ovis*

Temporal cluster*	Canonical Pathway	p-value^A^
***Cluster 1***	*p38 MAPK signalling*	*2.9E^-05^*
	IL12 signalling and production in macrophages	5.4E^-05^
	IL10 signalling	6.9E^-05^
	Aryl hydrocarbon receptor signalling	1.3E^-04^
	PPAR signalling	2.9E^-04^
***Cluster 2***	*IL10 signalling*	*1.2E^-10^*
	Production of nitric oxide and reactive oxygen species in macrophages	1.0E^-09^
	Dendritic cell maturation	1.8E^-09^
	TREM1 signalling	1.8E^-09^
	NF-kB signalling	6.7E^-09^
***Cluster 3***	*T helper cell differentiation*	*6.4E^-08^*
	IL10 signalling	3.7E^-06^
	NF-kB signalling	1.6E^-05^
	Acute phase response signalling	6.1E^-05^
	TREM1 signalling	2.4E^-04^
***Cluster 4***	*Interferon signalling*	*1.3E^-05^*
	Leukocyte extravasation signalling	2.9E^-05^
	Antigen presentation pathway	3.9E^-05^
	T helper cell differentiation	1.7E^-04^
	CD28 signalling in T helper cells	1.9E^-04^
***Cluster 6***	*Complement system*	*5.3E^-12^*
	Role of pattern recognition receptors in recognition of bacteria and viruses	2.9E^-06^
	Acute phase response signalling	8.7E^-05^
	Role of macrophages, fibroblasts and endothelial cells in RA^#^	1.0E^-03^
	Wnt/beta-catenin signalling	2.3E^-02^
***Cluster 7***	*Aryl hydrocarbon receptor signalling*	*3.5E^-03^*
	Antiproliferative role of somatostatin receptor 2	7.1E^-03^
	CCR5 signalling in macrophages	3.8E^-02^
	Integrin signalling	5.8E^-02^
	IL1 signalling	7.7E^-02^
***Cluster 8***	*Tight junction signalling*	*2.4E^-03^*
	BMP signalling pathway	2.3E^-02^
	TGF beta signalling	2.8E^-02^
	IL6 signalling	3.8E^-02^
	Wnt/beta-catenin signalling	5.1E^-02^

Key - *Cluster 5 contained too few genes for effective analysis of canonical pathways. ^#^Rheumatoid arthritis. ^A^p-value calculated using Fisher's exact test determining the probability that association between genes in the data set and canonical pathway is due to chance alone

#### Genes up-regulated with infestation, gene expression clusters 1-4

Cluster 1 - One hour post infestation

**Figure 3:**
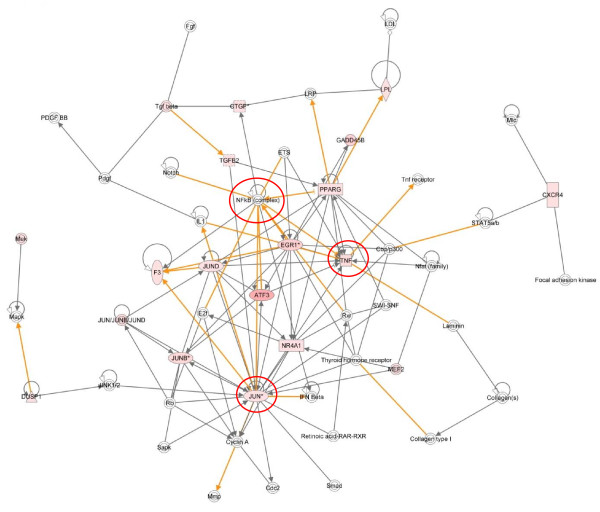
**IPA network depicting relationships among transcription factor encoding genes in cluster 1, 1 hour post-infestation with *P. ovis***. Merged representation of the two highest scoring networks. Individual nodes represent protein functions with relationships represented by edges. Nodes coloured by gene expression, red indicating up-regulation and white indicating gene/factor not differentially expressed but with defined relationship to other genes in network. Arrows indicate directional relationships. Red circles highlight roles for *JUN*, NF-kB and *TNF*. Only direct interactions selected

Cluster 1 represents the immediate early (IE) group of genes with peak expression at 1 hpi. The top biological function represented was "cancer" with 34 of the 51 pathway-eligible genes in this cluster associated with this category. The enrichment for cancer related genes is related to the abundance of transcription factors represented in this cluster and to highlight this, the top 2 networks from the Ingenuity pathway analysis were combined, forming a single network highly enriched for transcriptional regulators (Figure 3). This combined network demonstrates key roles for tumour necrosis factor alpha (TNFα), NF-kB and JUN each forming distinct hubs in the network (see red circles, Figure 3). A number of the genes in this network are known to be transcriptionally regulated by NF-kB, playing an important role in the instigation of the pro-inflammatory response. Further classification of cluster 1 using the DAVID Pathway Analysis software [19,20] identified enrichment for factors in the toll-like receptor (TLR) pathway. This was supported by IPA which identified that six genes from cluster 1 are members of the TLR signalling pathway (*FOS, JUN, JUNB, JUND, ATF3 *and *MAP2K6*). Indeed a number of genes from network 1 are classified as primary response genes for bacterial lipopolysaccharide (LPS), i.e. *FOS, JUN, TNF, EGR1 *and *NR4A1 *(Figure 3) [21,22].

The NF-kB family members *NFKB1*, *NFKB2 *and *RELB *and the NF-kB inhibitor complex factors *NFKBIA*, *NFKBIB*, *NFKBIE *and *NFKBIZ *were up-regulated following infestation with sheep scab with expression levels peaking at 3 hpi before declining towards baseline levels by 24 hours. It is well recognised that NF-kB is activated following signalling through the TLRs [23] (e.g. TLR4), to investigate this we mapped the temporal gene expression changes within the TLR signalling pathway following infestation. At 3 hpi, six genes (*MYD88, NFKB1, NFKB2, NFKBIA, TLR4 *and *TLR2*) from within the TLR signalling pathway showed peak expression (cluster 2). At 6 hpi (cluster 3) a further three members of this pathway showed peak expression (*CD14, EIF2AK2 *and *MYD88*) and at 24 hpi (cluster 4) peak expression of a further two genes (*IRAK4 *and *TLR6*) was observed. This indicates that early interactions between mites and host skin may lead to activation of elements of the TLR pathway, in turn leading to NF-kB activation. These findings are supported by a recent study by Trompette *et al*., [24] which demonstrated that the HDM allergen Der p 2 was capable of functional mimicry of lymphocyte antigen 96 (*LY96 *or *MD-2*) an integral part of the TLR4 signalling complex involved in the cellular response to bacterial LPS [25]. In addition, the *P. ovis *antigen Pso o 2, which shows a high level of sequence similarity with Der p 2 [26], instigates an NF-kB mediated pro-inflammatory response in ovine keratinocytes *in vitro *(S.T.G Burgess, unpublished observations).

Due to the increase in IL12 production following stimulation of APCs with LPS, TLR4 signalling has been associated with a Th1-type response [27]. However, TLR4 has been shown to be important in the induction of Th2 mediated allergic responses and for an ability to induce DC2 maturation (dendritic cells that support a Th2-type response) following exposure to helminth antigen [28]. Eisenbarth *et al*., [29] showed that the level of LPS exposure determines the type of inflammatory response, with exposure to high levels of LPS triggering a Th1 type response and exposure to low levels of LPS leading to Th2 type responses. It is likely that in a sheep scab infection occurring on the surface of the skin, that low levels of LPS from commensal gram negative bacteria would be present on the host skin. In addition, Hogg *et al*., [30] using 16S rRNA analysis demonstrated the presence of a number of bacterial species associated with *P. ovis*, including the gram negative bacterium *Serratia marcescens*, as such it is possible that LPS (derived from either host skin or mites) is present at the site of infection, and in conjunction with mite-derived allergens could trigger TLR4 instigating a pro-inflammatory response. Finally, Hammad *et al*., [31], demonstrated that HDM extract induced asthma via a TLR4-mediated mechanism resulting in activation of the Th2 promoting cytokines thymic stromal lymphopoeitin (TSLP), CSF2 (granulocyte-macrophage colony stimulating factor (GM-CSF)), IL25 and IL33. IL25, IL33 and TSLP were not represented on the ovine microarray used in this study and therefore we were unable to determine their expression levels. However *CSF2 *was activated within 1 hpi (9-fold increase) and these cytokines (*IL33, IL25, TSLP *&*CSF2*) have previously been linked to allergic immunopathology [32-34].

TNFα is up-regulated in human epithelial cells following exposure to HDM allergens and has also been detected in early stage lesions in sheep scab [11,35]. In support of these previous findings *TNF *was up-regulated (3.4-fold) in our dataset within 1 hpi, a response that declined by 3 hpi. TNFα, a pro-inflammatory cytokine the expression of which is controlled by NF-kB [36], is involved in cellular proliferation, differentiation and apoptosis and is also a potent pyrogen via stimulation of IL1 secretion [37]. Another factor of interest up-regulated at 1 hpi is tachykinin 1 (*TAC1 *or substance P, 2.3-fold). *TAC1 *is a neurotransmitter involved in induction of behavioural responses and as a vasodilator and secretagogue with well characterised roles in the itch response [38]. Up-regulation of *TAC1 *within the first hour of exposure could explain the rapid itching response observed with sheep scab [11].

In summary cluster 1 highlights the transcriptional response in the instigation of the host pro-inflammatory reaction to *P. ovis *infestation within the first hour. A number of key transcriptional regulators are involved in this reaction including NF-kB, AP-1, EGR1-3, TNFα and ATF3. The key signalling event, which is likely to be instigated by a mite-derived factor, closely mirrors that of the host response to LPS, potentially acting via a TLR to trigger NF-kB.

Cluster 2 - Three hours post infestation

**Figure 4:**
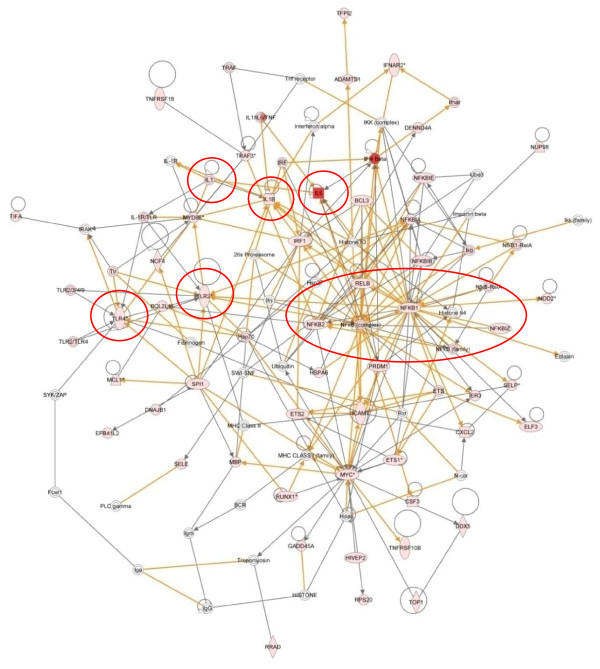
**IPA network depicting relationships among pro-inflammatory genes in cluster 2, 3 hours post-infestation with *P. ovis***. Merged representation of the four highest scoring networks. Individual nodes represent protein functions with relationships represented by edges. Nodes coloured by gene expression, red indicating up-regulation and white indicating gene/factor not differentially expressed but with defined relationship to other genes in network. Arrows indicate directional relationships. Red circles highlight roles for *IL1A*, *IL1B*, *IL6*, NF-kB, *TLR2 *and *TLR4*. Only direct interactions selected

Cluster 2 represents the early (E) group of genes showing a peak expression at 3 hpi. Pathway analysis identified the rapidly expanding host immune response with 62 of the 124 (50%) genes in this cluster associated with "inflammatory disease". The merging of the top 4 networks from the analysis of cluster 2 highlighted the up-regulation of NF-kB and pro-inflammatory genes (Figure 4). To enable the identification of the main hub factors this figure highlights only the direct interactions (Figure 4). Key roles for *TLR2 *(4.6-fold up), *TLR4 *(2.3-fold up), NF-kB and the pro-inflammatory cytokines *IL1A *(3-fold up), *IL1B *(15-fold up) and *IL6 *were identified (see red circles and oval, Figure 4), and are supported by enrichment for the "TLR" (p = 4.18E^-06^), "NF-kB" (p = 6.69E^-09^), "IL1" (p = 1.54E^-06^) and "IL6" (p = 8.09E^-08^) signalling pathways (Table 4). IL6 is a potent inducer of the acute phase response, with well characterised roles in a number of inflammatory diseases and is involved in a number of immune signalling pathways, i.e. TLR and IL10 signalling pathways [39]. *IL6 *demonstrated a 100-fold up-regulation at 1 hpi and a peak expression level of nearly 800-fold by 3 hpi before declining back to 15-fold up-regulated by 24 hpi. The implications for the sizeable and early up-regulation of *IL6 *following exposure to *P. ovis *are clear with the ability of this factor to induce acute phase protein synthesis, T-cell activation, stimulation of B cell Ig production and proliferation of keratinocytes [39]. This final point is of interest as sheep scab lesions are characterised by hyperkeratosis, evident by the presence of crusty scabs at the site of infection [7]. A similar up-regulation of *IL6 *has been observed *in vitro *in human monocyte and epithelial cells following exposure to the HDM allergen, Der p 1 [40,41]. The expression pattern of *IL6 *was mirrored by that of suppressor of cytokine signalling 3 (*SOCS3*), a well characterised inhibitor of IL6 signalling [42]. Like *IL6*, *SOCS3 *expression peaked at 3 hpi and then declined towards baseline levels by 24 hpi. It is plausible that *SOCS3 *is co-activated with *IL6 *as part of a negative feedback mechanism, possibly acting to control the level of *IL6 *expression induced following infestation with *P. ovis*. Seki *et al*., [43] demonstrated that SOCS3 regulates the onset and maintenance of Th2-mediated allergic responses and that this expression correlated with the pathology of the Th2-mediated diseases atopic dermatitis and asthma. SOCS3 is a potent negative regulator of interferon gamma (IFN-γ) signalling in keratinocytes and may play a role in the polarisation of immune responses towards Th2 via suppression of IFN-γ production [44].

IL1-α and IL1-β are pro-inflammatory cytokines proteolytically processed to form potent mediators of inflammation [45]. IL1 is one of the main participants in the initiation of inflammation and is important in the pathogenesis of skin diseases such as psoriasis and atopic dermatitis [46-48]. As well as being up-regulated here, IL1α and IL1β protein levels are increased in human skin equivalents stimulated with the scabies mites *S. scabiei *[49]. IL1 is expressed and stored intracellularly in keratinocytes, being quickly released in response to infection or injury [45]. Once released, IL1 triggers expression of prostaglandins, i.e. cyclooxygenase-2 (*COX-2 *or *PTGS2*, also differentially regulated here, 15-fold up at 3 hpi) and a number of selectin molecules, guiding chemotaxis of immune cells to the site of infection [45]. IL1 also induces expression of the chemokine *IL8 *which was highly up-regulated here (temporal cluster 4 and Table 3) and is likely to result in activation of surrounding keratinocytes, release of growth factors, such as fibroblast growth factor 7 (*FGF7*, 2.8-fold up at 24 hpi) and *CSF2 *(10-fold up between 1-6 hpi), and stimulation of keratinocyte proliferation [50,51]. Interestingly, extracts from *S. scabiei *have been shown to reduce IL8 production in keratinocytes, indicating the presence of differing responses to infestation with these two related mite species [52]. Suppression of *IL8 *by *S. scabiei *may be explained by the fact that unlike *P. ovis*, *S. scabiei *is a burrowing mite forming a more intimate interaction with the host than that observed with *P. ovis *which does not penetrate deeper than the stratum corneum [53]. Therefore *S. scabiei *may have greater need to evade the host immune response, unlike *P. ovis *which appears to rely on it as a food source. The IL1 surface receptor *IL1R1 *was also up-regulated here (2.2-fold, peaking at 6 hpi). This receptor binds both IL1α and IL1β leading to the activation of NF-kB, AP-1, MYC and EGR1 (all of which were up-regulated between 1 and 3 hpi) and the subsequent instigation of a pro-inflammatory response. The receptors *IL1R2 *(3.3-fold) and *IL1RN *(3.6-fold) were up-regulated at 6 hpi; these factors act as decoy receptors, dampening the response to IL1 through negative feedback mechanisms [54,55]. IL1 activity is thought to be controlled by IL4 via its ability to induce the expression of IL1R2 [55]. These mechanisms may act to attenuate the effects of IL1 at the site of infestation and could be linked to the increasing Th2 type response through IL4 induction.

As well as peak expression for a number of genes involved in TLR and NF-kB signalling pathways, cluster 2 included genes involved in the instigation of the host inflammatory response. The expression levels of selectin-E (*SELE*, 7-fold up) and selectin-P (*SELP*, average 18-fold up) both peaked at 3 hpi whilst selectin-L (*SELL*, 12-fold up) expression peaked at 6 hpi. Selectins are cellular adhesion molecules with roles in initiating leukocyte rolling along the endothelial barrier prior to intercellular adhesion molecule-1 (*ICAM1*) mediated adhesion and extravasation. Expression of *ICAM1 *(9-fold) also peaked at 3 hpi, falling back towards baseline levels by 24 hpi. The neutrophil chemo-attractant molecule *IL8 *showed a biphasic pattern of expression with one peak of up-regulation at 3 hpi (average 300-fold up), followed by a brief hiatus at 6 hpi (average 230-fold up) before finally peaking at 24 hpi (average 450-fold up). Biphasic patterns of *IL8 *mRNA expression have been observed following LPS stimulation of bovine alveolar macrophages and human white blood cells with initial peaks in mRNA expression between 1-4 hours followed by second peaks between 6-24 hours [56,57]. These studies demonstrated that the first wave of IL8 expression was due to the initial stimulation with LPS, whilst the secondary phase was due to local production of TNFα and IL-1 [56,57]. Our results support these findings with *IL1A, IL1B *and *TNF *all upregulated between 1-3 hours post-infestation due to stimulation by mite derived factors and then a second wave of *IL8 *expression likely to be stimulated by this initial pro-inflammatory response. A biphasic pattern of *IL8 *mRNA and protein expression has also been demonstrated in afferent lymph following infection of sheep skin with the ectoparasite *L. cuprinia*, indicating that upregulation of *IL8 *mRNA may also be reflected at the protein level [58]. The rapid increase in expression of factors involved in immune cell extravasation, i.e. selectins and adhesion molecules, at the site of infestation with *P. ovis *has obvious implications for disease progression, especially when combined with the significant up-regulation of chemo-attractant molecules like *IL8 *(300-fold), *IL6 *(700-fold up), colony stimulating factor 3 (*CSF3*, 18-fold up), *CSF2 *(GM-CSF, 10-fold up), chemokine (C-C) ligand 2 (*CCL2 *or MCP-1, 36-fold up) and chemokine (C-X-C) ligand 2 (*CXCL2 *or *GRO2*, 23-fold up).

*CCL2 *is chemo-attractant for monocytes and basophils, but not for neutrophils and eosinophils, and has previously been shown to be involved in the pathogenesis of psoriasis and atopic dermatitis [59]. Previous studies have demonstrated similar responses to HDM antigen, including up-regulation of adhesion molecules and cytokines, such as *CSF2, ICAM1, IL6 *and *CCL2 *[60]. *CXCL2 *was up-regulated in our dataset and has been shown to be chemotactic for neutrophils and to be involved in modulation of skin inflammatory responses [61,62]. A recent study also demonstrated the up-regulation of the related chemokine *CXCL1 *in human keratinocytes following exposure to HDMs, indicating a conserved response [63]. Sheep scab is characterised by a pronounced dermal oedema dominated by infiltration of eosinophils, accompanied by macrophages, lymphocytes, plasma cells, neutrophils and mast cells [11,12,64]. Therefore these findings help us to further understand lesion development, elucidating the mechanisms by which a local inflammatory response in the skin results in the recruitment and activation of circulating and skin resident immune cells. In contrast to these findings *S. scabiei *has been shown to down regulate the expression of *SELE *and this combined with the down regulation of IL8 by *S. scabiei *has been hypothesised to contribute to inhibition of neutrophil extravasation in early scabies lesions and may be related to immune evasion [65].

Mite-derived proteases, including serine, cysteine and aspartic proteases trigger host inflammatory responses [10,66]. A number of these act by triggering the protease activated receptors (PARs) leading to release of chemokines, such as IL8 and CCL2 and expression of the selectins *SELE *and *SELP *and *ICAM1 *[67-69]. Probes for the PAR family members were absent on the ovine microarray in this study so we were unable to determine any changes in their expression. However evidence points to their involvement as other components of these signalling cascades were up-regulated during the time course of infestation with *P. ovis *and homologues of the HDM allergens involved in the triggering of PARs have also been identified in *P. ovis*, i.e. Der p 1, Der p 3 and Der p 9 [67-70].

Sheep scab is classically associated with a Th2 type response, supported by infiltration of CD4^+ ^T-cells and γδ-T-cells, combined with a lack of CD8^+ ^T-cell infiltration and an influx of eosinophils and mast cells [71]. As well as induction of a number of pro-inflammatory factors with peak expression at 3 hpi, we detected a number of pro-allergic factors, in particular those involved in the development of a Th2 type response: *IL4 *(9-fold up) and the IL4 receptor (*IL4R*, 6-fold up) showed peak expression at 3 hpi and combined with a lack of up-regulation of the Th1-inducing factors, [IFN-γ, IL12 and IL18] this supports the Th2-based nature of sheep scab. These findings are combined with the up-regulation of IL18 binding protein (*IL18BP*, 8-fold at 6-hpi) which sequesters IL18, further inhibiting IL18 and IFN-γ production and also with the up-regulation of the canonical Th2 marker, *IL13 *(20-fold at 6 hpi). IL4 is released from mast cells and basophils following exposure to Der p 1, pointing to a link between activation of the innate immune system and the subsequent activation of an adaptive immune response [72].

In summary cluster 2 highlights the gathering pace of the pro-inflammatory response to infestation with *P. ovis*, likely to be triggered by the pro-inflammatory transcription factors identified in cluster 1. This is supported by up-regulation of pro-inflammatory cytokines such as *IL1A, IL1B, IL8 *and *IL6 *and increased expression of selectins and adhesion molecules, all of which are proposed to be involved in migration of immune cells to the site of infestation in sheep scab. These events may further exacerbate the early pro-inflammatory response, leading to oedema and serous exudate production, providing a ready source of food for the mites. These early changes in gene expression are in accord with an early pro-inflammatory reaction, followed by a Th2 associated pro-allergic response, laying the foundations for the allergic nature of sheep scab pathogenesis.

Cluster 3 - Six hours post infestation

Cluster 3 represents the intermediate (IM) group of genes with peak expression at 6 hpi. As in cluster 2, a number of these genes (91 of the 207 pathway mapping eligible genes in this cluster) are involved in inflammatory disease, along with "inflammatory response" (80/207), "cell-cell signalling and interaction" (79/207), "immunological disease" (81/207) and "cellular growth and proliferation" (104/207). The main canonical pathway represented here was "T-helper cell differentiation" (p = 6.38E^-08^), followed by the "IL10" (p = 3.72E^-06^), "NF-kB" (p = 1.57E^-05^), and "acute phase response" signalling pathways (p = 6.13E^-05^) and "communication between innate and adaptive immune cells" (p = 2.6E^-04^) (Table 4). Development of a pro-allergic Th2 type response appears to be a critical step in disease progression in sheep scab. Analysis of the genes enriched within the canonical pathway for "T-helper cell differentiation" supports this, with increased expression of *CD86 *(6-fold), *IL2RG *(6-fold), *IL2RA *(11-fold), *IL10 *(2.8-fold) and *IL13 *(20-fold).

**Figure 5:**
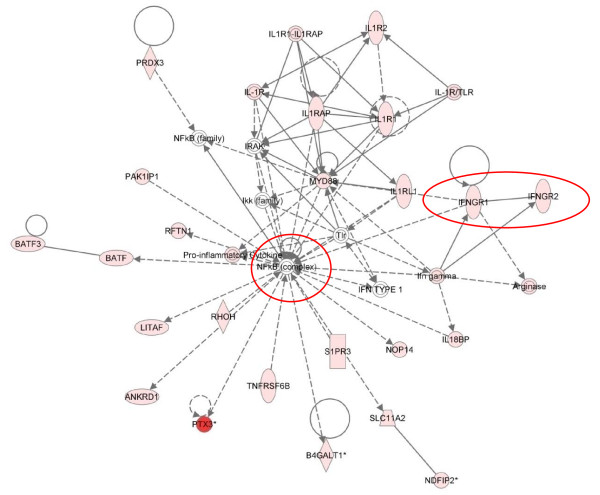
**IPA network depicting relationships among pro-inflammatory genes in cluster 3, 6 hours post-infestation with *P. ovis***. Individual nodes represent protein functions with relationships represented by edges. Nodes coloured by gene expression, red indicating up-regulation and white indicating gene/factor not differentially expressed but with defined relationship to other genes in network. Arrows indicate directional relationships. Red circles highlight roles for NF-kB, *IFNGR1 *and *IFNGR2*. Network score = 38.

**Figure 6:**
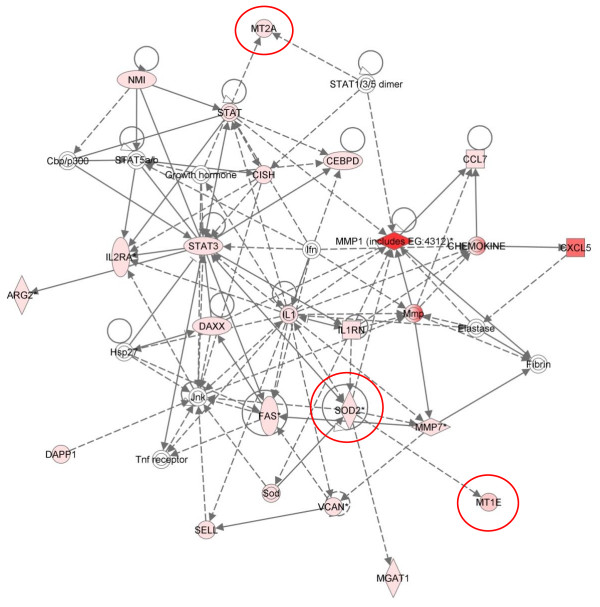
**IPA network depicting relationships among genes in cluster 3, 6 hours post-infestation with *P. ovis***. Individual nodes represent protein functions with relationships represented by edges. Nodes coloured by gene expression, red indicating up-regulation and white indicating gene/factor not differentially expressed but with defined relationship to other genes in network. Arrows indicate directional relationships. Red circles highlight roles for *MT1E, MT2A *and *SOD*. Network score = 31.

Network 2 from cluster 3, showed enrichment (network score = 38) for genes involved in infectious and dermatological disease (Figure 5). This network highlighted the role of IL1 signalling at 6 hpi and the pivotal role played by NF-kB in this process is clear (see red circle, Figure 5). Increased expression of specific IFN-γ receptor genes, *IFNGR1 *and *IFNGR2*, can also be seen from this network (see red oval, Figure 5). However, in the absence of increased expression of IFN-γ, along with the skewing towards a Th2 type response it is probable that their expression is indicative of a general T-cell activation. This network also highlights the activation of pentraxin 3 (*PTX3*) which may be induced by an increase in local IL1β [73]. PTX3 is a soluble pattern recognition receptor (PRR) activated by inflammatory signals and also by TLR activation [74] and has roles in modulating the inflammatory response, interacting with complement component C1Q, enabling it to either activate or inhibit the complement cascade [75]. *PTX3 *is up-regulated in H292 cells exposed to HDM extract, indicating a conserved role in sheep scab pathogenesis [17]. *PTX3 *expression is down-regulated by the Th2 cytokines IL4 and IL13 and, in our dataset, *PTX3 *expression begins to decline by 24 hpi with an average 60-fold increase in expression compared to the average 270-fold peak at 6 hpi and coinciding with the peak up-regulation of *IL13 *at 6 hpi [76] and matrix metalloproteinase 1 (*MMP1*, average 270-fold up) which is itself regulated by IL1β [77]. The expression of *MMP1 *is induced by TNFα, and IL6 is released in response to cytokine signalling in skin subjected to UV-induced DNA damage [78,79]. MMP1, also known as fibroblast collagenase, hydrolyses collagen fibrils in the extracellular matrix of the dermis, with important implications in sheep scab pathogenesis [80]. Other MMPs, notably *MMP7 *(3-fold) and *MMP13 *(2.3 fold), were up-regulated at 6 hpi. MMPs have been implicated in other allergic diseases, for example *MMP9 *is the predominant MMP in asthma and its expression is associated with enhanced airway inflammation [81]. Additional genes clustering with *PTX3 *in a sub-cluster within cluster 3 were superoxide dismutase 2 (*SOD2*, 30-fold up), metallothionein 1E (*MT1E*, 77-fold up) and metallothionein 2A (*MT2A*, 10-fold up). The acute phase protein SOD2 is activated in response to oxidative stress acting to destroy toxic radicals, protecting cells from toxic oxygen, its anti-oxidant functions have also been linked to its anti-inflammatory properties [82]. Metallothioneins have a cytoprotective role following the instigation of an inflammatory response, and can protect against allergic airway inflammation induced by ovalbumin (OVA) by suppression of *IL1B *expression [83]. This cluster of genes may be indicative of an orchestrated attempt to modulate the excessive inflammatory response induced by the presence of mite factors and may inform novel methods by which sheep scab could be controlled. These interactions can be observed in more detail in cluster 3, network 4 (see red ovals, Figure 6).

The TNF superfamily member, *CD40 *was up-regulated with peak expression (6.5-fold) at 6 hpi and a 5.4-fold induction at 3 hpi. The HDM allergen Der p 1 proteolytically cleaves CD40, rendering dendritic cells (DCs) less responsive to signalling through the CD40-CD40L pathway [84]. DCs matured in the presence of Der p 1 also induced production of less IFNG and IL12 and more IL4 from CD4+ T-cells, directing a Th2 type response to mite exposure [84]. *CSF2 *(*GM-CSF*), although not classified as significantly differentially expressed in our dataset did show a strong up-regulation following infestation, confirmed by qRT-PCR (9-fold up at 1 and 3 hpi, peaking at 10-fold by 6 hpi). *CSF2 *is over-expressed by keratinocytes in atopic dermatitis lesions and its release is up-regulated from keratinocytes stimulated with Der p 1 [85,86]. Pastore *et al*., [87] showed that this up-regulation was due to the dysregulated activation of the transcription factor AP-1 (composed of JUN and FOS both up-regulated at 1 hpi). It is plausible that the up-regulation of *FOS *and *JUN *at 1 hpi with *P. ovis *could lead to increased expression of *CSF2*. CSF2 has been shown to be essential for DC development and is involved in the regulation of DC function; this could have important implications for sheep scab pathogenesis, by reducing DC function and interfering with antigen presentation by DCs [88]. Expression of *IL10 *peaked at 6hpi (2.8-fold). IL10 is an anti-inflammatory cytokine with roles in NF-kB inhibition, prevention of Th1 T-cell clonal expansion, activation of mast cells and Th2 T-cells [89-91]. The up-regulation of *IL10 *may be indicative of a concerted effort to dampen Th1 responses, further biasing the immune system towards a Th2 pro-allergic response and may influence the down-regulation of the NF-kB genes found in cluster 2 by 6 hpi.

IL2 receptor alpha (*IL2RA*) showed peak expression at 6 hpi (11-fold up), indicative of an influx of immune cells at the site of infestation with *P. ovis*. IL2RA (also known as CD25) has been shown to be enzymatically cleaved by Der p 1 and promotes development of allergic inflammatory responses [92]. Cleavage of IL2RA by Der p 1 resulted in diminished proliferation of T-cells and a reduction in IFN-γ secretion [92], presumably promoting a bias towards a Th2-type response due to the impaired proliferation of Th1-type cells. This is further supported by the observations that Der p 1 fails to impair T-cell proliferation in response to exogenous IL4 in mice, promoting a Th2-type response by modulating IL4 and IFN-γ [93,94]. Lee *et al*., [95] previously characterised a *P. ovis *orthologue of Der p 1, termed Pso o 1 and it is possible that this may act in a similar manner by enzymatically cleaving IL2RA.

In summary, peak expression of the Th2 cytokines *IL13 *and *IL4 *in cluster 3 highlights the continuing bias towards a Th2 based immune response to infestation with *P. ovis *and shows the up-regulation of a number of factors involved in wound repair and dampening of the inflammatory response. Cluster 3 highlights methods by which mite-allergens may modulate the host immune response, for example through the cleavage of immune factors, i.e. CD25.

Cluster 4 - Twenty four hours post infestation

Cluster 4 represents the late (L) genes with peak up-regulation at 24 hpi. The top biological function represented was "inflammatory response" with 89 of the 235 genes associated with this category. A number of additional biological functions including, "cell-cell signalling and interaction" (86/235), "cellular growth and proliferation" (111/235) and "cellular movement" (65/235) were enriched. Enriched canonical pathways included "leukocyte extravasation signalling" (p = 2.96E^-05^), "antigen presentation" (p = 3.9E^-05^), "T-helper cell differentiation" (p = 1.74E^-04^), "dendritic cell maturation" (p = 5.96E^-04^) and "IL8 signalling" (p = 3.1E^-03^) (Table 4). The largest fold change was observed for *IL8 *(600-fold induction) which represented its peak expression level. IL8 is a potent chemo-attractant and angiogenic factor involved in the chemotaxis of neutrophils, basophils and T-cells, and has also been shown to be involved in eosinophil chemotaxis [96]. Increased *IL8 *expression supports our previous findings from ovine keratinocyte cultures stimulated with *P. ovis *mite wash and whole mite extract [14]. However, the timing of this response differs between the *in vitro *and *in vivo *systems as the previous study had shown that *IL8 *expression levels began to decline by 24 hpi; whereas this time point represented the peak level *in vivo *[14]. However, it cannot be ruled out that this response continues to be activated beyond 24 hpi. This difference in timing may arise due to additional signals provided by immune cells present *in vivo *in the skin layer absent in the *in vitro *ovine keratinocyte cultures. Similar responses to those seen in this study with IL8 have also been observed in epithelial cells treated with HDM extract [17,49].

Another cytokine with peak expression at 24 hpi was *regakine *(8-fold) which acts in synergy with either C5a or IL8 (both of which were up-regulated in our dataset) to recruit circulating leukocytes into inflamed tissue [97,98]. This finding has implications for sheep scab which is characterised by high levels of the pro-inflammatory cytokine IL8 [14], and it is plausible that IL8 and regakine could work synergistically with other cytokines to enhance the inflammatory response to infestation with *P. ovis*. In addition, as described for cluster 6 (below), the HDM derived allergen Der p 3 cleaves complement components C3 and C5, producing the anaphylatoxins C3a and C5a, respectively [99]. Local production of IL8 could combine with the anaphylatoxin C5a, derived from allergen-mediated cleavage of C5, and with regakine acting synergistically to produce a more pronounced inflammatory response. *Regakine *has also been shown to be up-regulated in cattle skin infected with the tick *Rhipicephalus (Boophilus) microplus *[100]. Co-activation of C5a, regakine and IL8 by the recently identified Der p 3 homologue, Pso o 3 (S.T.G. Burgess, unpublished data) following infestation with *P. ovis *could potentially lead to increased levels of inflammation via enhanced migration of inflammatory cells to the site of infestation, further exacerbating disease pathogenesis [97,98].

**Figure 7:**
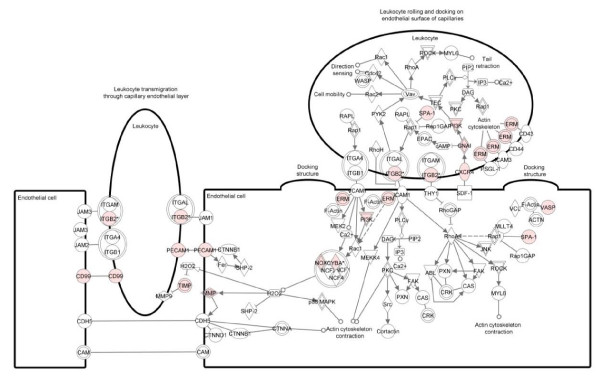
**IPA representation of the canonical pathway for leukocyte extravasation signalling in cluster 4, 24 hours post-infestation with *P. ovis***. Individual nodes represent protein functions with relationships represented by edges. Nodes coloured by gene expression, red indicating up-regulation and white indicating gene/factor not differentially expressed but with defined relationship to other genes in network. Arrows indicate directional relationships

**Figure 8:**
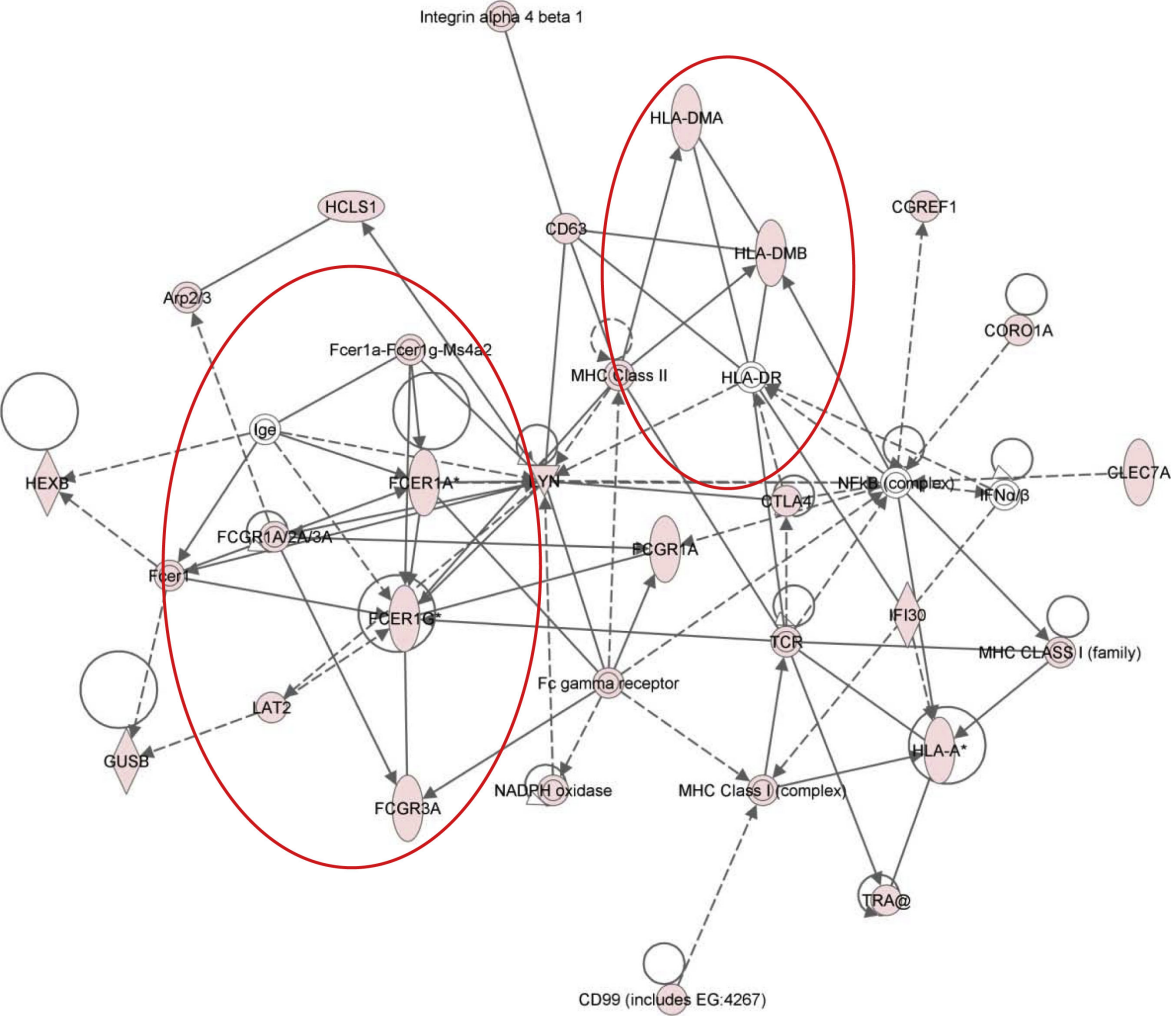
**IPA representation of IgE and pro-Th2 genes in network 3 from cluster 4, 24 hours post-infestation with *P. ovis***. Individual nodes represent protein functions with relationships represented by edges. Nodes coloured by gene expression, red indicating up-regulation and white indicating gene/factor not differentially expressed but with defined relationship to other genes in network. Arrows indicate directional relationships. Red circles highlight roles for *FCER1A*, *FCER1G, FCGR3A, FCGR1A *and selected MHC complex genes. Network score = 31.

Figure 7 shows the canonical pathway for leukocyte extravasation overlaid with the up-regulated genes from cluster 4 and shows an increase in many of the factors involved, indicating the strong influence of this activity at 24 hpi. These findings are consistent with previous reports highlighting a rapid influx of immune cells at the site of infestation with *P. ovis *[7] and the expression of a number of adhesion molecules, such as selectins, E, L and P at 3-6 hpi as described here. Consistent with our findings at 3-6 hpi there was also a significant enrichment for factors involved in "T-helper cell differentiation" towards a Th2 type response (p = 1.74E^-04^). An additional pathway highlighted at this stage was "dendritic cell maturation" (p = 5.96E^-04^). Ten molecules (*CD86*, *FCER1G, FCGR1A, FCGR3A, HLA-A, HLA-DMA, HLA-DMB, IRF8, PIK3CD *and *TRA@*) from this pathway were up-regulated at 24 hpi and elements of this pathway overlap closely with network 3, cluster 4 highlighting the induction of an IgE based pro-allergic Th2-type response to infestation with *P. ovis *at this time point (see red ovals, Figure 8).

Cytotoxic T lymphocyte associated protein 4 (*CTLA4*) showed peak up-regulation (7.7-fold) at 24 hpi (Figure 8). CTLA4 is a cell surface marker, associated with T-regulatory (T-reg) cell function, which binds to CD80 and CD86 on antigen presenting cells (APCs) sequestering them and preventing their interaction with CD28 on T-cells [101-103]. T-reg cells may be involved in sheep scab pathogenesis with the infiltration of T-reg cells in lesional skin observed within 2 weeks of infestation with *P. ovis *[104]. Although probes for the T-reg cell marker *FOXP3 *were absent from the ovine microarray used here we observed a 1.8-fold increase in its expression at 3 hpi, rising to 2.4-fold by 6 hpi as determined by qRT-PCR, offering support for the potential involvement of T-reg cells in sheep scab pathogenesis. Figure 8 highlights the increased expression (3.7-fold) of the C-type lectin molecule *CLEC7A *(Dectin-1) at 24 hpi. Further C-type lectins were up-regulated at this time with increased expression of *CLEC4E *(Mincle) (26-fold), *CLEC4A *(DCIR) (6.4-fold), *CLEC4G *(6-fold), *CLEC6A *(Dectin-2) (5-fold), *CLEC2D *(4.4-fold) and the mannose receptor C type 1 (*MRC1 *or *CD206*) (3.3-fold). C-type lectins are non-toll PRRs involved in recognition of pathogen associated molecular patterns (PAMPs) [105]. They have roles in the migration of DCs and in determining the outcome of their interactions with lymphocytes [106].

A previous study demonstrated the involvement of MRC1 in the uptake of Der p 1 in human monocyte derived DCs [107]. This interaction was more efficient in HDM-allergic patients, suggesting that its involvement in pathogenesis of allergic disease [107]. Dectin-1 is a receptor for beta-glucan and this interaction is activated in respiratory epithelial cells following exposure to HDM extracts [108]. These findings are supported by Kobayashi *et al*., [61], who showed that activation of keratinocytes by fungal beta-glucan resulted in activation of Dectin-1 and also the non-toll PRR, *NOD2 *(up-regulated here, 2.2-fold at 3 hpi). Therefore activation of Dectin-1, along with additional C-type lectins and other non-toll PRRs, i.e. NOD2, by mite derived allergens, combined with activation of TLR4 by HDM Der p 2 [(cluster 2, above) and as described by Trompette *et al*., [24]] could trigger activation of DCs, providing a link between the innate and adaptive immune systems in sheep scab. The activation of innate immune factors, such as IL1A, IL1B, TNFα, CSF2 and IL8 (all up-regulated here within 24 hpi) activates cutaneous acquired immunity by enhancing antigen presentation on DCs [109]. In addition, Der p 1 has been shown to cleave the C-type lectins DC-SIGN and DC-SIGNR, resulting in loss of cell surface DC-SIGN and reduced ICAM3 binding [110]. ICAM3 is involved in the trafficking of DCs and in particular with polarisation towards a Th1 based immune response, thus reduced binding of ICAM3 following mite allergen-mediated cleavage of DC-SIGN and DC-SIGNR could further bias towards a Th2 based immune response to *P. ovis *[111,112]. Genes encoding the major histocompatibility complex factors, *HLA-A, HLA-DMA *and *HLA-DMB *are highlighted in Figure 8, with peak expression at 24 hpi supporting the enrichment of antigen presentation. Figure 8 also shows the up-regulation of a number of genes involved in antibody dependent responses, namely the Fc fragment of IgE receptors, *FCER1A *(3-fold up), *FCER1G *(5.7-fold up) and the Fc fragment of IgG receptors, *FCGR1A *(CD64, 5.4-fold up) and *FCGR3A *(CD16, 4.7-fold up). The Fc gamma receptors, FCGR1A and FCGR3A are activated by immune complexes involving IgG and are involved in maturation of DCs via triggering of IL10 production [113,114]. These molecules can trigger production of ICAM1, CD86 and CD40, involved in further propagation of the immune response [84,92,115], and were also up-regulated here. FCER1A and FCER1G are IgE receptors that play key roles in allergic diseases by coupling allergen with mast cells initiating inflammatory and immediate hypersensitivity responses [113,116]. This results in release of inflammatory mediators and secretion of lymphokines, furthering the development of the local inflammatory reaction [113]. Although the expression of these Fc receptors peaks at 24 hpi they are up-regulated by greater than 2-fold by 3-6 hpi in our dataset. The implications of the increased expression of these receptors provides further indication of progression towards a Th2-type response at the site of infestation and highlights the speed at which these events may unfold. Therefore the up-regulation of these factors (with the exception of IL5) shown here within the first 24 hours is indicative of early activation of a Th2-mediated pro-allergic response in sheep scab.

Cluster 4 contains genes involved in terminal differentiation of keratinocytes, with the small proline rich proteins (SPRRs) *SPRR2A *(324-fold up) and *SPRR2E *(average 136-fold up) showing high levels of expression. In addition the S100 calcium binding proteins, *S100A9 *(90-fold) and *S100A12 *(66-fold) were up-regulated. These genes are components of the epidermal differentiation complex (EDC), a genomic locus encoding for proteins involved in terminal differentiation of keratinocytes and ensuring integrity of skin barrier function [117]. A similar up-regulation of these genes has been observed in psoriasis and atopic dermatitis, and as in this study these changes were accompanied by the down-regulation of other EDC genes such as loricrin and filaggrin (see cluster 8 for further details (below), Sugiura *et al*., [118]). SPRRs are predominantly expressed in squamous epithelium, where they contribute to the formation of the cornified envelope, providing structural integrity and reducing permeability [119]. They have previously been shown to be up-regulated in response to epidermal injury (i.e. UV radiation) and in response to pro-inflammatory molecules e.g. IL1 [120,121]. In addition, Zimmermann *et al*., [122] showed that SPRR2A and SPRR2B are involved in regulation of allergic inflammation and that they are induced by IL13 in experimental allergic responses.

The up-regulation of SPRRs in scab infected skin is likely to be controlled by the inflammatory response, either through the action of IL1, IL13, or both. The up-regulation of EDC genes at this time may aid skin barrier remodelling in an attempt to reconstruct an effective barrier against exogenous factors following mite and/or mite allergen-mediated damage. In relation to this, previous studies have highlighted the ability of HDM allergens (Der p 1 and Der p 3), for which *P. ovis *homologues have been identified (Pso o 1 and Pso o 3, respectively) to cleave intercellular tight junctions, thus opening up the tight junction barrier and increasing epithelial permeability [123,124]. The proteolytic activity of Der p 1 also reduces skin barrier function in nude mice [125], disrupting the critical barrier function of the skin mediated by the stratum corneum [126] increasing the risk of further allergen sensitisation [125].

The S100 factors S100A9 and S100A12 (90-fold and 66-fold up at 24 hpi) represent two of fourteen S100 factors encoded for within the EDC [127]. *S100A2*, an oxidative stress-regulated factor over-expressed in psoriasis [128], was also up-regulated here however its expression peaked at 6 hpi (2.6-fold). S100A9 is a stress induced factor which, together with S100A8, forms the heterodimeric complex calprotectin [129]. *S100A9 *is expressed at low levels in normal skin, however like *S100A2 *and *S100A12*; these factors are both highly expressed in psoriatic and atopic skin lesions [130-132]. Like S100A9, S100A8 is a marker of keratinocyte activation and has roles in wound healing and inflammatory cell chemoattraction [132], in addition, S100A9 displays anti-microbial activity and has a role in resistance to invasion by pathogenic bacteria [133]. It also acts as a pro-inflammatory mediator, up-regulating IL8 release and surface expression of ICAM1 [134]. As well as local release from keratinocytes, S100A8 and S100A9 are produced by circulating neutrophils further enhancing inflammatory responses by influencing local trafficking of leukocytes [135]. Importantly, S100A9 up-regulates transcription of genes under NF-kB control enhancing development of endotoxic shock in response to LPS [136]. Ehrchen *et al*., [137] demonstrated that calprotectin amplifies innate immunity by triggering TLR4, thus further promoting the inflammatory response and enhancing extravasation of leukocytes to the site of inflammation. The S100 proteins S100A8 and S100A9 play roles in the hyper-proliferation and abnormal differentiation of keratinocytes observed in psoriasis, an effect that is mirrored in the pathogenesis of sheep scab with the development of severe crusted lesions [7,11,138].

The increased expression of S100 genes in scab infected skin could lead to exacerbation of an already prolific inflammatory response.

In summary, cluster 4 highlights the activation and maturation of DCs, indicating that the initial pro-inflammatory response is beginning to influence an adaptive immune response to *P. ovis *infestation. This cluster demonstrates that this is biased towards a Th2 based pro-allergic response to infestation. Cluster 4 also shows the increased involvement of antigen presentation and leukocyte extravasation. It is clear that the early pro-inflammatory response observed in sheep scab develops into a Th2, pro-allergic response to the presence of mites which is more likely to lead to disease exacerbation than to effective parasite clearance.

#### Genes down-regulated with infestation, gene expression clusters 5-8

Cluster 5 - One hour post infestation

Cluster 5 represents the immediate early (IE) repressed genes with peak repressed expression at 1 hpi. There were insufficient genes in this cluster for effective pathway mapping. However, five genes were down-regulated at 1 hpi, namely apolipoprotein B mRNA editing enzyme, catalytic polypeptide-like 2 (*APOBEC2*), zinc activated ligand-gated ion channel (*ZACN*), heat shock 70kDa protein 8 (*HSPA8*), ADAM metallopeptidase with thrombospondin type 1 motif, 2 (*ADAMTS2*) and fibrinogen-like 2 (*FGL2*).

Cluster 6 - Three hours post infestation

**Figure 9:**
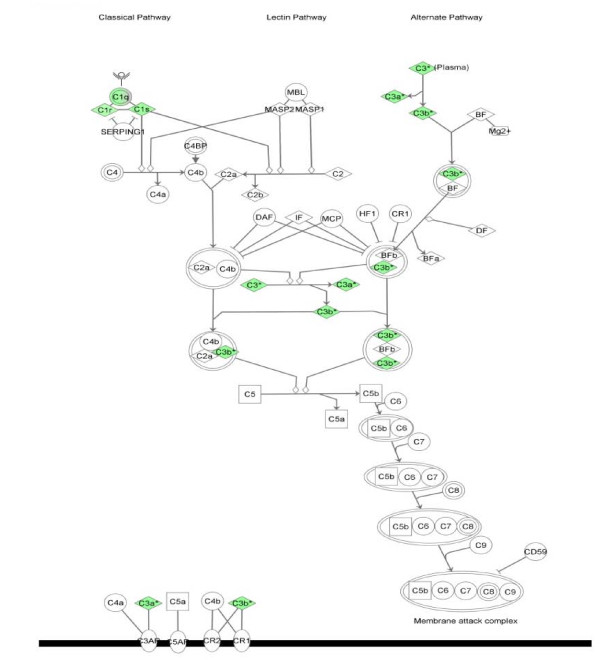
**IPA representation of the complement system pathway enriched in cluster 6, 3 hours post-infestation with *P. ovis***. Individual nodes represent protein functions with relationships represented by edges. Nodes coloured by gene expression, green indicating down-regulation and white indicating gene/factor not differentially expressed but with defined relationship to other genes in network. Arrows indicate directional relationships

Cluster 6 represents the early (E) repressed genes with peak repressed expression at 3 hpi. The biological functions enriched in cluster 6 were "infectious disease" (6 of the 19 pathway mapping eligible genes), "connective tissue disorders" (5/19), "dermatological disease and conditions" (4/19) and "antigen presentation" (2/19). This cluster showed enrichment for the complement system (p = 5.29^-12^), with six complement genes (*C3, C1QA, C1QB, C1QC, C1R *and *C1S*) down-regulated at 3 hpi (Table 4). This is highlighted in Figure 9, which shows the components of the complement pathway regulated at this time.

The complement genes (*C3, C1QA, C1QB, C1QC, C1R *and *C1S*) clustered together, sharing a very similar expression profile. These genes have a repressed level of expression between 1-6 hpi. However, by 24 hpi their expression had returned to pre-infestation levels (between 1-2 fold up-regulated), indicating the potential presence of a transcriptional control mechanism to repress complement in the early stages of infestation with *P. ovis*. Interestingly, Vroling *et al*., [17] also observed a down-regulation of *C3 *in airway epithelial cells following exposure to HDM extract. The complement system is a well characterised target of the HDM cysteine protease Der p 1 which proteolytically cleaves complement components C3 and C5 resulting in production of the anaphylatoxins C3a and C5a and formation of the membrane attack complex [99]. C3a and C5a are biologically active peptides involved in recruitment and activation of DCs, eosinophils and mast cells [139]. C3a and C5a are pro-allergic and their generation could further influence the immune response in the direction of Th2. In addition to their pro-inflammatory properties, the production of C5a and C3a may, through exogenous proteases, result in down-regulation of complement components at the transcriptional level as part of a negative feedback mechanism, limiting the potentially damaging effects of high levels of anaphylatoxin. As discussed earlier, high levels of *IL8 *and *regakine *could act in synergy with C5a to further amplify the inflammatory response. The gene encoding the C5a receptor, *C5AR*, was also up-regulated 10-fold by 3 hpi, remaining high at over 7-fold up-regulated at 24 hpi. C5AR is involved in promotion of immune cell recruitment and inflammation and intriguingly cross-talk between this pathway and the TLR pathways, notably TLR4 and TLR2, has been reported [140,141].

A family of inactivated serine protease paralogs, termed scabies mite-inactivated protease paralogs (SMIPPs), has been identified in the scabies mite *S. scabiei *[142]. These factors inhibit the human complement system by binding to molecules involved in separate arms of the complement cascade, C1q (classical pathway), mannose binding lectin (lectin pathway) and properdin (complement factor P (CFP), alternative pathway) [142,143]. Inhibition of these factors is proposed to allow scabies mites to limit host complement production, thus limiting complement-mediated gut damage and creating a favourable micro-environment for mite survival [142,143]. The genes encoding the three subunits that make up C1q, namely *C1QA, C1QB *and *C1QC *were down-regulated at 3 hpi here, indicating a potential inhibitory effect on the classical complement pathway in sheep scab infestation. However no change in expression was observed for mannose binding lectin and conversely *CFP *was up-regulated (6-fold) by 24 hpi, indicating a potentially positive effect on the alternative complement pathway in sheep scab. To date no *P. ovis *homologues of the *S. scabiei *SMIPPs have been identified, however EST data from the sheep scab mite remains limited.

In summary cluster 6 highlights the reduced expression of complement genes following infestation with *P. ovis*. The mechanism for this remains unclear however it is conceivable that mites specifically target the complement pathway to evade this potentially damaging host response or in order to further exacerbate the localised immune response.

Cluster 7 - Six hours post infestation

Cluster 7 represents the intermediate (IM) repressed genes with peak down-regulated expression at 6 hpi. The top biological function represented was "cancer", (78 of the 150 pathway mapping eligible genes) indicating a general down regulation in gene expression. Additional enriched biological functions included "cellular movement" (46/150), "gastrointestinal disease" (30/150) and "cellular growth and proliferation" (64/150). This cluster is dominated by genes encoding collagens, including *COL4A1, COL5A2, COL1A2, COL1A1 *and *COL3A1*. In a similar pattern to complement genes, these genes are repressed between 1-6 hpi with expression levels returning towards pre-infestation levels by 24 hpi. *P. ovis *and HDM extracts contain proteases capable of collagen degradation [144,145]. In addition, interstitial collagenase (*MMP1*) which was 270-fold up-regulated at 6 hpi is involved in collagen breakdown in the extra-cellular matrix and is also accompanied by the increased expression of its inhibitory factor *TIMP1 *(6.5-fold up-regulated) at this time. Suppression of collagen related genes could be in response to elevated levels of inflammatory activity or as part of a general remodelling/wound healing event in response to infestation. Separation and disruption of collagen bundles has been observed in sheep skin following *P. ovis *infestation, indicating a role for collagen disruption in disease pathogenesis [11].

In summary cluster 7 highlights the reduced expression of a number of collagen genes at 6 hpi with *P. ovis*. The repressed expression of these genes may be part of a host strategy to affect localised tissue repair and wound healing, however it may also be due to the requirement for increased tissue access for immune cells extravasating to the skin.

Cluster 8 - Twenty four hours post infestation

The final cluster of genes contained the late (L) repressed genes with peak repressed expression at 24 hpi and as with cluster 4 (24 hpi up-regulated) represents the end stage of the time course. The top biological function represented was "cancer" (60 of the 109 pathway mapping eligible genes). Additional biological functions associated with this cluster included "cell death" (44/109), "dermatological diseases and conditions" (12/109) and "cellular growth and proliferation" (45/109). As observed with cluster 4, cluster 8 was enriched for genes involved in terminal differentiation of keratinocytes with a number of genes located within the EDC locus. The most repressed of these genes were loricrin (*LOR*, 26-fold down-regulated), filaggrin (*FLG*, 5.8-fold down), S100 calcium binding protein A3 (*S100A3*, 2-fold down) and late cornified envelope 1B (*LCE1B*, 2-fold down), however a further three EDC-associated genes; PDZ domain containing 1 (*PDZK1*); selenium binding protein 1 (*SELENBP1*) and endosulfine alpha (*ENSA*) were repressed 2-fold, at 24 hpi.

Down regulation of filaggrin expression is interesting from the perspective of mite proteases, in particular for the serine protease Der p 3 found in HDM extracts. A homologue of Der p 3 has been identified in *S. scabiei *(Sar s 3) and is capable of cleaving recombinant human filaggrin *in vitro *[146]. Using an antibody specific to human filaggrin the authors were able to demonstrate the presence of human filaggrin within the scabies mite gut, indicating active ingestion of this protein [146]. As stated earlier we recently identified a *P. ovis *homologue of Der p 3, termed Pso o 3, although the serine protease activity of this allergen has yet to be confirmed (S.T.G. Burgess, unpublished observations). Digestion of filaggrin by mite proteases could damage the epidermal barrier, reducing skin barrier function and rendering the epidermis susceptible to external factors, such as allergens and secondary infections, a scenario likely to be important in disease pathogenesis [7].

The mechanisms responsible for down-regulation of filaggrin and loricrin at this stage are unclear. However, expression of these factors, along with the involucrin (*IVL*), which was also down-regulated here (1.98-fold between control and 24 hpi) have been shown to be down-regulated through the suppressive action of Th2 cytokines, such as IL4 and IL13 [147,148]. It is possible that this mechanism exists to prevent further cell differentiation in response to wound healing and the expanding inflammatory response. Previous studies on atopic dermatitis have shown that defective skin barrier function allows allergen penetration and initiates immunological reactions [149]. The altered expression of EDC genes and defective epidermal differentiation are implicated in the pathogenesis of other skin and allergic diseases, including atopic dermatitis, ichthyosis vulgaris, psoriasis and asthma and these changes in expression are linked to mutations and single nucleotide polymorphisms (SNPs) within EDC genes [150-154]. It is currently believed that loss-of-function mutations in EDC genes, such as filaggrin, enable increased allergen penetration and therefore heighten the risk of allergen sensitisation [155]. These findings could potentially lead to the identification of host genes responsible for the observed innate susceptibility or resistance of different breeds of sheep to *P. ovis *infestation. Loricrin, filaggrin and involucrin are crucial components of the epidermal barrier and their down-regulation in scab infected skin, potentially due to the increase in Th2 cytokine expression, may lead to further barrier dysfunction, increasing mite allergen exposure and further exacerbating pathogenesis. The reduced expression of filaggrin, loricrin and involucrin has also been observed in human atopic dermatitis skin lesions and, as found here, this was accompanied by up-regulation of the S100 calcium binding proteins *S100A2 *and *S100A9 *(see cluster 4 and also [118]).

Also down regulated (2-fold) at 24 hpi was claudin-3 (*CLDN3*); the claudins are involved in the formation of tight junctions in the epithelium and claudin-1 is enzymatically cleaved by Der p 1 resulting in tight junction barrier disruption [123]. In addition "tight junction signalling" (p = 2.35E^-03^) was amongst the top canonical pathways associated with cluster 8 (Table 4), with the expression of six genes from this pathway repressed (*ACTC1, CLDN3, CNKSR3, JUN, MYL1 *and *TIAM1*). The disruption of tight junction signalling may be crucial in pathogenesis of sheep scab where allergen penetration beyond the stratum corneum would be dependent on their enzymatic breakdown.

A number of keratin encoding genes showed an approximate 2-fold down-regulation at 24 hpi. These included keratin 2 (*KRT2*), *KRT34*, *KRT71*, *KRT83 *and genes encoding keratin-associated proteins (KRTAP), namely *KRTAP1-1*, *KRTAP12-2*, *KRTAP13-1*, *KRTAP16-1, KRTAP24-1, KRTAP6-1, KRTAP6-2, KRTAP7-1, KRTAP8-1 *and *KRTAP8-2*. Keratin 1 (*KRT1*, 7-fold down) and the gene encoding keratin-associated protein (*KRTAP16-3*, 4.4-fold down) were more highly repressed and this repression began at an earlier stage (2-fold repressed at 1 hpi) peaking at 24 hpi. The down-regulation of these genes following infestation with *P. ovis *may result in further perturbation of the skin barrier and could be a response to the increasing inflammatory reaction and mechanical skin damage. Interestingly, KRT1 is a predicted target for digestion by the *S. scabiei *allergen, Sar s 3 (a homologue of Der p 3 (HDM) and of Pso o 3 from *P. ovis*), if this proves to be the case, the implications for skin barrier function following this digestion may be similar to those expected with the down-regulation of filaggrin [146].

In summary cluster 8 highlights the importance of EDC genes and combined with the up-regulation of other EDC genes within cluster 4 (also 24 hpi), indicates an important role for this gene complex in sheep scab pathogenesis. Other roles of note within this cluster are the reduced expression of genes involved in the formation of tight junctions and also for keratin and keratin-associated genes.

## Conclusions

This paper represents the first transcriptome level analysis of the host skin response to infestation with *P. ovis*. A combined network/pathway based approach has enabled the elucidation of the temporal patterns of gene expression through which the host response is regulated. As such, a clearer picture has emerged of the mechanisms by which mites and mite-derived factors trigger a pro-inflammatory reaction in the skin. An intact epithelial barrier is crucial for maintenance of skin defence, preventing penetration of allergens beyond the stratum corneum and from making contact with APCs in sub-epithelial tissues [125]. Amongst others, *P. ovis *produces homologues of the HDM proteolytic enzymes Der p 1 and Der p 3 which may be involved in the initial interactions with host skin via proteolytic disruption of the epidermal barrier. This digestion may enable mite antigens to enter the sub-epidermal layer and interact with APCs, leading to allergic sensitisation. These initial host-parasite interactions trigger a pro-inflammatory response leading to expression of cytokines, chemokines, selectins and adhesion molecules and to activation and extravasation of leukocytes at the site of infestation. Combined with the activation of dermal DCs these events bias the immune response towards an allergic Th2 type profile, involving T-cells, B-cells and IgE production. The IgE produced binds to and activates mast cells in the skin, instigating the release of further inflammatory factors. During the early stages of infestation this Th2 type response is likely to be beneficial to the mites, providing them with a serous exudate as a food source and appears to have no detrimental effect on mite survival. This Th2 response may also be involved in the down-regulation of EDC genes, further exacerbating disease progression by limiting skin barrier function recovery, allowing further interactions to occur between mite antigens and host APCs and establishing a vicious cycle of continued antigen exposure and immune response. This study has begun to unravel the mechanisms by which *P. ovis *instigates such a profound pro-inflammatory and pro-allergic response in the host whilst simultaneously identifying the inflammatory/immune pathways involved, it has also highlighted clear parallels between the host response to *P. ovis *in sheep and the response of a variety of hosts to a number of similar mite species, i.e. HDMs and *S. scabiei*, further supported by the histopathological similarities between sheep scab lesions and skin lesions in atopic dermatitis [12]. As such, the data presented here may have important implications for a number of diseases affecting livestock, domesticated animals and humans such as atopic dermatitis, asthma, psoriasis and sarcoptic mange. The information described here will also be invaluable for the identification of novel methods of disease control.

## Methods

### Animal study

Ethical approval for this study was obtained from the Moredun Research Institute Experiments Committee and animals monitored daily in accordance with guidelines agreed with UK Home Office. *P. ovis *mites (mixed population consisting of adults, nymphs and larvae) were harvested from infected donor animals maintained at the Moredun Research Institute. Sheep scab naïve, 1-2 year old Scotch mule lambs (n = 6) were maintained at the Moredun Research Institute. Two days prior to infestation with *P. ovis *mites, an area of the left flank (50 cm (long) × 20 cm (deep)) of each animal was shaved to the skin with electric clippers and six plastic isolation chambers (1 cm deep × 1 cm diameter) were adhered to the skin with surgical glue (Indermil, UK). After one hour to allow glue drying and chamber adhesion, six skin biopsies were removed from each animal from within the isolation chambers using a disposable 8 mm biopsy punch (Dunlops, UK), following the administration of a local anaesthetic 1-2 ml, 2% (w/v) lignocaine hydrochloride and 0.001% (w/v) adrenaline (Lignol, Arnolds, Harlescott, UK). Following biopsy removal sampling sites were sealed with Michel surgical suture clips (Dunlops, UK). Skin biopsies were placed into 5 ml RNA Later solution (Ambion, UK) and stored at +4°C overnight before transfer to -20°C. These samples constituted the non-infected samples per animal (six biopsies per animal for six animals). For the time course samples, eight further isolation chambers were adhered to the shaven area of the left flank of each animal as described above. Approximately 20-50 mites were placed directly onto the skin within each chamber and mites were then carefully removed with a cotton bud after the following time intervals, 1, 3, 6 and 24 hpi (two chambers corresponding to each time point). Following mite removal a skin biopsy was taken from within each chamber as detailed above and stored in RNA Later as described above.

### Total RNA extraction

RNA was extracted from skin biopsies following the random assignment of samples to limit batch effects. Biopsies were removed from RNA Later and excess solution and contaminating blood removed. Biopsies were dissected with a clean scalpel into smaller sections and placed into a bead homogeniser tube (2 ml CK14 type, Precellys, UK) with 1 ml of lysis buffer (Buffer RLT with β-mercaptoethanol (Qiagen, UK) and lysed with 2 cycles [6200 rpm for 23 seconds] in a bead homogeniser (Precellys 24, Precellys UK), holding on ice for 2 minutes between cycles. Lysate was cleared by centrifugation [13,000 rpm at +4°C] and supernatant transferred to a microcentrifuge tube. Total RNA was extracted with the RNeasy fibrous tissue midi-kit (Qiagen, UK) following the manufacturer's protocol with on-column DNase I digestion. RNA sample quality was assessed on an Agilent Bioanalyser (Agilent, UK), an RNA Integrity Number (RIN) was obtained for each sample and RNA yield was assessed on a ND-1000 Nanodrop spectrophotometer (Thermo Scientific, UK). RNA samples with a RIN >7.5 were considered to be of acceptable quality [15]. Duplicate RNA samples from each time point, per animal were pooled (5 μg RNA from each of 2 replicates for 1, 3, 6 and 24 hpi samples, forming 10 μg pools per time point/animal) and also for the non-infected samples (5 μg RNA from each of 6 replicates for non-infected samples forming 30 μg pools per animal).

### Microarray study

Transcriptomic analysis was performed with the Agilent ovine gene expression microarray (15,208 ovine probes) in an 8 × 15K slide format. Samples were randomly assigned to the arrays within a slide, with all samples (time points) for a single animal hybridised onto the arrays on a single slide to limit technical variation. Total RNA was used as starting material for each array with one array used per time point (×5) (non-infected (time = 0), 1, 3, 6 and 24 hpi), per animal with a total of 30 arrays. The Agilent One-Colour gene-expression workflow (Cy3 dye, Quick Amp Labelling Protocol, Agilent, UK) was used to amplify and process the RNA samples, following the manufacturer's protocols. Briefly 800 ng total RNA was used for the generation of fluorescently labelled (Cy3 dye) complementary RNA (cRNA), using T7 RNA polymerase. Microarrays were hybridised at 65°C for 17 hours in a hybridisation oven (Agilent, UK). Microarrays were scanned on a microarray scanner (Agilent, UK) at the manufacturer's recommended settings. Microarray signal data was extracted using Agilent Feature Extraction software version 9.5.3 (Agilent, UK). To enable inter-array comparisons raw microarray data for each array was normalised to the 75^th ^percentile and log transformed, further downstream filtering was performed in Genespring GX 11.0 (Agilent, UK).

### Statistical analysis of microarray data

Differential gene expression across the time course of infestation was determined using a one way-analysis of variance (ANOVA) with a Student-Newman-Keuls (SNK) post-hoc test in Genespring GX 11.0 (Agilent Technologies, UK) comparing each of the 5 time points, non-infected (time = 0), 1, 3, 6 and 24 hpi across all animals. Multiple test correction was performed using the Benjamini & Hochberg False Discovery Rate (FDR) procedure with an FDR corrected p-value cut-off of ≤ 0.05 and a fold change cut-off of ≥2.0 [156].

### Pathway analysis

Data were analysed with Ingenuity Pathways Analysis (IPA) (Ingenuity Systems, http://www.ingenuity.com). Gene clusters identified from the time course analysis and clustering described formed the input data set. Each gene identifier was mapped to its corresponding gene object in Ingenuity's Knowledge Base. Gene networks were then algorithmically generated based on their connectivity and assigned a score (a numerical value used to rank networks according to how relevant they are to the genes in the input dataset). IPA uses a right-tailed Fisher's test to calculate the p-value for networks. A score of 10 indicates a p = 10^-10 ^chance that genes in that network are associated solely by chance. A network is a graphical representation of the molecular relationships between molecules, molecules being represented as nodes and relationship between nodes being represented as edges. All edges are supported by at least 1 reference from the literature. Networks were analysed to identify the biological functions and/or diseases most significant to the genes in that network. Canonical pathway analysis identified the biological pathways of most significance. The significance of the association between the data set and canonical pathway was determined based on two parameters: (1) Ratio of the number of genes from the data set that map to the pathway divided by the total number of genes that map to the canonical pathway and (2) p-value calculated using Fisher's exact test determining the probability that the association between the genes in the data set and the canonical pathway is due to chance alone.

### qPCR validation

Quantitative real-time PCR (qRT-PCR) was used to verify differential expression of 11 selected genes from the final list of differentially expressed genes. Primer sets were designed as previously described (See Additional file 2, Table S1 for primer sequences) [157,158]. Briefly, a two-step qRT-PCR was performed using the standard curve method. Plasmids and standard curves for the selected genes and the endogenous control glyceraldehyde 3-phosphate dehydrogenase (*GAPDH*) were prepared as previously described [157,158]. Total RNA was used as template for the generation of complementary DNA (cDNA) using Superscript II (Invitrogen, UK) and anchored oligo(dT) primers (Sigma, UK) according to the manufacturer's protocols. Serial 1:10 dilutions of plasmid containing the gene of interest ranging from 10^8 ^to 10^2 ^copies per μl were run in parallel with each series of samples on an ABI Prism 7000 Sequence Detection System (Applied Biosystems, UK), allowing automatic standard curve generation. PCR efficiencies calculated from the slopes were consistently ≥90% for all of the qRT-PCR assays performed. The number of copies per μl of sample was calculated and results normalized to the *GAPDH *endogenous control, the expression of which had been shown not to vary significantly with infestation based on the Agilent ovine microarray data (Data not shown). Samples and standards were run in triplicate and melting curve analysis was performed at the end of each PCR to verify product specificity. Genes were selected to represent a number of the clusters identified in the time course analysis to provide a range of genes expressed over different time points. In addition the *FOXP3 *gene which was not present on the Agilent ovine expression microarray, but known to be important in regulation of host responses to sheep scab infestation, was also analysed. Finally, qRT-PCR analysis was performed on the *CSF2 *and *IL4 *genes as these appeared to be differentially expressed on the arrays but their probe sets failed on flags for certain samples. Expression profiles of selected genes were analysed over the 24 hours of infestation and compared to the microarray results (where applicable).

## Authors' contributions

STGB designed the study, organised the research trial, performed and processed samples and microarray hybridizations, data analysis, clustering and pathway/network analysis using IPA, qPCR validations and wrote the manuscript. DF participated in the research trial and qPCR validations. FN participated in the research trial. CW participated in study design, research trial, EBI submission of the microarray data and helped prepare the manuscript. TNM contributed towards qPCR validations, data interpretation and helped prepare the manuscript. AJN participated in the study design and data analysis and helped prepare the manuscript. JFH conceived and designed the study, participated in the analysis and helped prepare the manuscript. All authors have read and approved the manuscript

## Supplementary Material

Additional file 1 **Gene lists for individual clusters**. The file contains the list of genes represented in each of the individual gene expression clusters described in the text. This file includes gene symbol annotation where available, Agilent probe ID, fold change data for each gene at each time point compared to baseline (Time = 0) and the Benjamini & Hochberg FDR corrected p-values for each individual probe. Click here for file

Additional file 2 **Table S1 - Primer sequences and annealing temperatures for qRT-PCR validation of microarray data**. The file contains the primer sequences and annealing temperatures for the qRT-PCR validation of the microarray data used in this study. Click here for file
