# Supplementary material for: Transcriptomic analysis of the temporal host response to skin infestation with the ectoparasitic mite Psoroptes ovis
Source: BMC Genomics. 2010 Nov 10;11:624. doi: 10.1186/1471-2164-11-624 (PMC3091762; doi:10.1186/1471-2164-11-624)
Supplement: Additional file 2 — Table S1 - Primer sequences and annealing temperatures for qRT-PCR validation of microarray data. The file contains the primer sequences and annealing temperatures for the qRT-PCR validation of the microarray data used in this study. [file 1471-2164-11-624-S2.DOC]

## Additional file 2 – Table S1 - Primer sequences and annealing temperatures for qRT-PCR validation of microarray data

| **Gene** | **Primer Type** | **Primer Sequence (5’→3’)** | **Product size (bp)** | **Annealing temp (°C)** |
| --- | --- | --- | --- | --- |
| *GAPDH^A^* | External  Internal | F, AAG GCA GAG AAC GGG AAG  R, AGT GAT GGC GTG GAC AGT  F, GGT GAT GCT GGT GCT GAG TA  R, TCA TAA GTC CCT CCA CGA TG | 366  265 | 55  57 |
| *CSF2* (*GM-CSF*)*^A^* | External  Internal | F, GCC TGC TTC ACT TCT GGA C  R, GCT TCT CCT GGG CAC TGT  F, GAT GGA TGA AAC AGT AGA AGT CG  R, CAG CAG TCA AAG GGA ATG AT | 425  261 | 55  57 |
| TNFα (*TNF*)*^A^* | External  Internal | F, TCC TTG GTG ATG GTT GGT  R, CAC TGA CGG GCT TTA CCT C  F, GAA TAC CTG GAC TAT GCC GA  R, CCT CAC TTC CCT ACA TCC CT | 525  238 | 58  57 |
| *TGFBI^A^* | External  Internal | F, GCC CTG GAC ACC AAC TAC TG  R, TCA GCT GCA CTT GCA GGA G  F, GAA CTG CTG TGT TCG TCA GC  R, GGT TGT GCT GGT TGT ACA GG | 338  169 | 61  55 |
| *IL1B^A^* | External  Internal | F, CTG TGT TCT TCC CTT CCC TT  R, CAA AAA TCC CTG GTG CTG  F, CCT TGG GTA TCA GGG ACA A  R, TGC GTA TGG CTT TCT TTA GG | 518  317 | 55  57 |
| *IL6^A^* | External  Internal | F, GCT TCC AAT CTG GGT TCA  R, CCA CAA TCA TGG GAG CCG  F, TCC AGA ACG AGT TTG AGG  R, CAT CCG AAT AGC TCT CAG | 347  236 | 55  52 |
| *IL8^A^* | External  Internal | F, GAA GTC CTC TGG GAC AGC AG  R, TTG GAA GCA ATG GAA AAA GG  F, ATG AGT ACA GAA CTT CGA  R, TCA TGG ATC TTG CTT CTC | 429  222 | 55  55 |
| *IL10^A^* | External  Internal | F, AGC TGT ACC CAC TTC CCA  R, GAA AAC GAT GAC AGC GCC  F, TGA AGG ACC AAC TGA ACA GC  R, TTC ACG TGC TCC TTG ATG TC | 305  160 | 55  55 |
| *IL18^A^* | External  Internal | F, TCA GAT CAC GTT TCC TCT CC  R, GAT GGT TAC AGC CAG ACC TC  F, GAG CAC AGG CAT AAA GAT GG  R, TGA ACA GTC AGA ATC AGG CAT A | 348  241 | 55  57 |
| *FOXP3^A#^* | Internal | F, GTG CAA TCT CTG GAG CAA CA  R, AAC CCT TGT CAG ATG ATG CC | 121 | 59 |
| *IL4^A*^* | Internal | F, AGA GAT CAT CAA AAC GCT GAA  R, GTC TGC TAC AGG CAG CTC | 70 | 57 |
| *TLR4^B^* | External  Internal | F, ATGATGGCGCGTGCC  R, GCGGCATTTACTTGTTAACTGAA  F, TGGATTTATCCAGATGCGAAA  R, GGCCACCAGCTTCTGTAAAC | 2598  152 | 57  57 |
| *TLR2^B^* | External  Internal | F, AATGCCACGTGCTTTGTG  R, CAC CACCAGACCAAGACTGA  F, GGCTGTAATCAGCGTGTTCA  R, GATCTCGTTGTCGGACAGGT | 2392  160 | 58  58 |

*^A^*Primers kindly supplied by Tom McNeilly as described in [157]. *^B^*Primers kindly supplied by Jung-Su Chang & George Russell as described in [158]. *Ovine *IL4* expression plasmid kindly supplied by Anne Wood, Moredun Research Institute.
